# Inhibition of Human Drug Transporter Activities by the Pyrethroid Pesticides Allethrin and Tetramethrin

**DOI:** 10.1371/journal.pone.0169480

**Published:** 2017-01-18

**Authors:** Lisa Chedik, Arnaud Bruyere, Marc Le Vee, Bruno Stieger, Claire Denizot, Yannick Parmentier, Sophie Potin, Olivier Fardel

**Affiliations:** 1 Institut de Recherches en Santé, Environnement et Travail (IRSET), UMR INSERM U1085, Faculté de Pharmacie, 2 Avenue du Pr Léon Bernard, Rennes, France; 2 Pôle Pharmacie, Centre Hospitalier Universitaire, 2 rue Henri Le Guilloux, Rennes, France; 3 Department of Clinical Pharmacology and Toxicology, University Hospital Zurich, University of Zurich, Rämistrasse 100, Zurich, Switzerland; 4 Centre de Pharmacocinétique, Technologie Servier, 25–27 rue Eugène Vignat, Orléans, France; 5 Pôle Biologie, Centre Hospitalier Universitaire, 2 rue Henri Le Guilloux, Rennes, France; Universite Paris Diderot, FRANCE

## Abstract

Pyrethroids are widely-used chemical insecticides, to which humans are commonly exposed, and known to alter functional expression of drug metabolizing enzymes. Limited data have additionally suggested that drug transporters, that constitute key-actors of the drug detoxification system, may also be targeted by pyrethroids. The present study was therefore designed to analyze the potential regulatory effects of these pesticides towards activities of main ATP-binding cassette (ABC) and solute carrier (SLC) drug transporters, using transporter-overexpressing cells. The pyrethroids allethrin and tetramethrin were found to inhibit various ABC and SLC drug transporters, including multidrug resistance-associated protein (MRP) 2, breast cancer resistance protein (BCRP), organic anion transporter polypeptide (OATP) 1B1, organic anion transporter (OAT) 3, multidrug and toxin extrusion transporter (MATE) 1, organic cation transporter (OCT) 1 and OCT2, with IC_50_ values however ranging from 2.6 μM (OCT1 inhibition by allethrin) to 77.6 μM (OAT3 inhibition by tetramethrin) and thus much higher than pyrethroid concentrations (in the nM range) reached in environmentally pyrethroid-exposed humans. By contrast, allethrin and tetramethrin *cis*-stimulated OATP2B1 activity and failed to alter activities of OATP1B3, OAT1 and MATE2-K, whereas P-glycoprotein activity was additionally moderately inhibited. Twelve other pyrethoids used at 100 μM did not block activities of the various investigated transporters, or only moderately inhibited some of them (inhibition by less than 50%). *In silico* analysis of structure-activity relationships next revealed that molecular parameters, including molecular weight and lipophilicity, are associated with transporter inhibition by allethrin/tetramethrin and successfully predicted transporter inhibition by the pyrethroids imiprothrin and prallethrin. Taken together, these data fully demonstrated that two pyrethoids, *i*.*e*., allethrin and tetramethrin, can act as regulators of the activity of various ABC and SLC drug transporters, but only when used at high and non-relevant concentrations, making unlikely any contribution of these transporter activity alterations to pyrethroid toxicity in environmentally exposed humans.

## Introduction

Pyrethroid insecticides constitute presently one of the most widespread class of synthetic pesticides [1]. These compounds are structurally divided into two types, *i*.*e*., type I pyrethroids containing a cyclopropane carboxylic acid structure, and type II pyrethroids having an α-cyano group attached to a benzylic carbon. They are commonly used in agriculture, in food storage and transportation and in indoor and outdoor residential settings. Humans are thought to be widely exposed to these pesticides [2, 3], especially through the diet [4], because pyrethroids are frequently detected in some fruits and vegetables [5]. Additionally, dusts and surfaces can contribute to pyrethroid exposure at residences [6], whereas occupational exposure to pyrethroids has also to be taken into account, notably in agricultural workers [7].

The insecticidal actions of pyrethroids depend on their ability to bind to voltage-gated sodium channels of insect nerves and to prevent their closure, leading by this way to permanent depolarization of axonal membranes and paralysis [8]. Pyrethroids can also impair mammal ionic channels, including voltage-gated sodium or calcium channels and voltage- and ligand-gated chloride channels [9]. Mammals, including humans, are however considered as rather poorly sensitive to toxic effects of pyrethroids, even if human poisoning can lead to severe neurotoxicity, resulting in tremor syndrome for type I pyrethroids and choreoathetosis with salivation syndrome for type II pyrethroids [10]. Chronic exposure to some pyrethroids may moreover result in developmental neurotoxicity and nigrostriatal dopaminergic neurodegeneration, thus favoring Parkinson's disease and other neurodegenerative diseases [11], that are also thought to be promoted by other chemical classes of pesticides [12]. Pyrethroids may additionally contribute in a notable way to deleterious effects of pesticide mixtures [13].

Besides ion channels, other molecular targets of pyrethroids have been described in mammals. It is notably the case for γ-aminobutyric acid receptors [14] and for several hormone receptors [15], which may contribute to endocrine disruption properties of pyrethroids [16]. Pyrethroids can also activate drug sensing receptors such as pregnane X receptor (PXR) [17] and constitutive androstane receptor (CAR) [18]; by this way, they have been shown to up-regulate expression and activity of some major drug metabolizing enzymes like cytochrome P-450 (CYP) 3A4 and CYP2B6 in human hepatic cells [19, 20], and thus to modulate drug metabolizing pathways [21]. In addition to CYPs, pyrethroids may impact mammalian drug transporters, which contribute in a major way to xenobiotic detoxification [22] and are often coordinately regulated with drug metabolizing enzymes [23]. Indeed, activity of P-glycoprotein (P-gp), an ATP-binding cassette (ABC) efflux pump encoded by the multidrug resistance (MDR) 1 gene/*ABCB1* and handling a broad range of amphiphilic cationic drugs as well as various endogenous lipids [24, 25], has been shown to be inhibited by some pyrethroids [26, 27]. The ABC efflux pump breast cancer resistance protein (BCRP/*ABCG2*) may also interfere with pyrethroids because its ATPase activity is blocked by the pyrethroids permethrin and cypermethrin [28]. Moreover, indirect evidence for the handling of the pyrethroids deltamethrin, *cis*-permethin and *trans*-permethrin by an uncharacterized influx transporter in intestinal Caco-2 cells has been recently reported [29]. Data about interactions of pyrethroids with human drug transporters remain however yet rather scarce and limited. The present study was therefore designed to more extensively analyze the potential inhibitory effects of fourteen pyrethroids towards activity of main ABC and solute carrier (SLC) drug transporters. These insecticides correspond to type I pyrethroids (seven compounds: allethrin (also known as allethrin 1), bifenthrin, *cis*-permethrin, *trans*-permethrin, resmethrin, tefluthrin and tetramethrin) or type II pyrethroids (seven compounds: β-cyfluthrin, λ-cyhalothrin, β-cypermethrin, deltamethrin, esfenvalerate, fenpropathrin and τ-fluvalinate) and can be considered as adequately reflecting the structural and historical diversity of these pesticides. Moreover, at least some of them are insecticides used in large-scale commercial agricultural and domestic applications and have already been tested in *in vitro* toxicity assays [30–33]. Our data demonstrate that the pyrethroids allethrin and tetramethrin are inhibitors of various drug transporters, but only when used at relative high concentrations likely not reached in humans environmentally exposed to these insecticides.

## Materials and Methods

### Chemicals

Pyrethroids were provided by Sigma-Aldrich (Saint-Quentin Fallavier, France) and Cluzeau Info Labo (Sainte-Foy-La-Grande, France). The chemical structures of the fourteen pyrethroids whose the potential inhibitory effects towards activity of drug transporters were extensively tested are shown in S1 Fig. It is noteworthy that pyrethroid insecticides generally have complex configurations and contain one to three chiral centers, thus resulting in two to eight stereoisomers, with only some of them displaying insecticide properties [9, 34, 35]. Most, if not all, of these insecticides can therefore be theoretically considered as mixtures of geometric and optical isomers, knowing however that some commercial preparations of pyrethroids available on the market may contain only one or some of possible stereoisomers [34]. Unfortunately, the exact composition and stereoisomer proportion of the pyrethroids used in the present study were not provided by the suppliers. The total number of possible stereoisomers for each of the fourteen pyrethroids extensively analyzed in the study is given in S1 Table. Pyrethroids were initially prepared as stock solutions (50 mM) in dimethyl sulfoxide. Such stock solutions were next dissolved in the transport assay medium described below, for getting working pyrethroid concentrations tested on transporter activities. Rhodamine 123, verapamil, probenecid, amitriptyline, fumitremorgin C, fluorescein, 4',6'-diamidino-2-phenylindole (DAPI), and tetra-ethylammonium bromide (TEA) were purchased by Sigma-Aldrich, whereas carboxy-2,7-dichlorofluorescein (CF) diacetate and Hoechst 33342 were from Life Technologies (Saint Aubin, France). [1-^14^C]-TEA (sp. act. 3.5 mCi/mmol), [6,7-^3^H(N)]-estrone-3-sulfate (E3S) (sp. act. 54 Ci/mmol) and 3,4-[Ring-2,5,6-^3^H]- dihydroxyphenylethylamine (dopamine) (sp. act. 46 Ci/mmol) were from Perkin-Elmer (Boston, MA, USA). All other chemicals were commercial products of the highest purity available.

### Cell culture

P-gp-overexpressing mammary MCF7R cells, parental MCF7 cells [36] and multidrug resistance-associated protein (MRP) 2/*ABCC2*-expressing human hepatoma HuH-7 cells [37] were cultured in Dulbecco’s modified Eagle medium (DMEM) (Life Technologies), supplemented with 10% (vol/vol) fetal calf serum, 20 IU/mL penicillin and 20 μg/mL streptomycin. BCRP-transfected HEK293 cells (HEK-BCRP cells) [38], kindly donated by Pr X. Decleves (Faculty of Pharmacy, University Paris-Descartes, Paris, France), were cultured in DMEM supplemented with 10% (vol/vol) fetal calf serum, 100 IU/mL amoxicillin, 100 μg/mL erythromycin and 2000 μg/mL G418. Organic anion transporter polypeptide (OATP) 1B1/*SLCO1B1*- and OATP1B3/*SLCO1B3*- transfected CHO cells (CHO-OATP1B1 and CHO-OATP1B3 cells) were cultured in DMEM-low glucose (1 g/L) containing 20 IU/mL penicillin, 20 μg/mL streptomycin, 10% (vol/vol) fetal calf serum, 50 μg/mL proline and 500 μg/mL G418, as previously reported [39]; CHO wild type cells were maintained in the cell medium, without however G418. HEK293 cells overexpressing organic cation transporter (OCT) 1/*SLC22A1* (HEK-OCT1 cells), OCT2/*SLC22A2* (HEK-OCT2 cells), multidrug and toxin extrusion transporter (MATE)1/*SLC47A1* (HEK-MATE1 cells), organic anion transporter (OAT) 1*/SLC22A6* (HEK-OAT1 cells), OAT3*/SLC22A8* (HEK-OAT3 cells) and OATP2B1*/SLCO2B1* (HEK-OATP2B1 cells) were prepared by transduction of HEK293 cells by lentiviral pLV-EF1-hOCT1-hPGK-GFP, pLV-EF1-hOCT2-hPGK-GFP, pLV-EF1-hMATE1-hPGK-GFP, pLV-EF1-hOAT1-hPGK-GFP, pLV-EF1-hOAT3-hPGK-GFP, pLV-EF1-hOATP2B1-hPGK-GFP or pLV-EF1-hNTCP-hPGK-GFP vector, as previously described [40]. Control HEK293 cells (HEK-MOCK cells) were obtained in parallel by transduction of an empty lentiviral PLV-EF1-hPGK-GFP vector. Construction of the lentiviral vectors, production of lentivirus supernatants, transduction of HEK293 cells, cloning and initial characterization of HEK-OCT1, HEK-OCT2, HEK-MATE1, HEK-OAT1, HEK-OAT3 and HEK-OATP2B1 cells were performed by Vectalys (Labège, France). Transduced HEK293 cells were next routinely cultured in DMEM medium supplemented with 10% (vol/vol) fetal calf serum, 20 IU/mL penicillin, 20 μg/mL streptomycin, 1% (vol/vol) MEM non-essential amino acids solution (Life Technologies) and 1 μg/mL insulin.

For transport assays, cells were usually seeded in 48-multiwell Falcon^TM^ tissue culture-treated polystyrene or Corning^TM^ BioCoat^TM^ poly-D-lysine plates (Corning Incorporated, NY, USA). The type of multiwell plates, the initial cell seeding and the number of culture days before performing transport assays are indicated for each cell line/clone in S2 Table.

### ABC and SLC transporter activity

The effects of pyrethroids on activity of ABC and SLC transporters were determined through measuring cellular accumulation or retention of fluorescent or radiolabeled reference substrates for transporters, in the presence or absence of reference inhibitors, as previously described [41]. The nature of cells and reference substrates and inhibitors used for transport assays are summarized in S3 Table.

For accumulation assays (performed for all transporters, excepted BCRP), transporter-expressing cells usually cultured in 48-well plates were first incubated at 37°C with reference substrates in the absence (control) or presence of pyrethroids or reference inhibitors, in a well-defined transport assay medium [42], consisting of 136 mM NaCl, 5.3 mM KCl, 1.1mM KH_2_PO_4_, 0.8 mM MgSO_4_, 1.8 mM CaCl_2_, 10 mM HEPES, 11 mM D-glucose and adjusted to pH = 7.4 (excepted for MATE transporter assays, for which pH was set to 8.4). The nature of substrates and reference inhibitors and the incubation times with substrates, that varied according to the transporter, were selected in agreement with previous studies [41, 43] and are indicated in S3 Table. The total incubation volume with substrates was 200 μL/well. After washing twice in phosphate-buffered saline (PBS) (200 μL/well/washing), cells were lysed in distilled water (155 μL/well). An aliquot of cell lysate (5 μL) was then taken for determining cellular protein content by the Bradford method [44]. The rest of lysate (150 μL/well) was used for measuring intracellular accumulation of reference substrates by scintillation counting or spectrofluorimetry using a SpectraMax Gemini SX spectrofluorometer (Molecular Devices, Sunnyvale, CA, USA) (excitation and emission wavelengths were 485 and 535 nm, respectively, for rhodamine 123 and fluorescein, and 355 and 460 nm, respectively, for DAPI).

For retention assay (used for BCRP activity), HEK-BCRP cells cultured in 48-well plates were first loaded at 37°C with transport assay (200 μL/well) containing 16.2 μM Hoechst 33342 for 30 min. After washing twice in PBS (200 μL/well/washing), cells were reincubated in Hoechst 33342-free transport assay medium (200 μL/well) at 37°C for 90 min in the absence or presence of pyrethroids or of the BCRP reference inhibitor fumitremorgin C used at 10 μM. Cells were next lysed in distilled water (155 μL/well) after washing twice in PBS (200 μL/well/washing). The cell lysate was finally used for determining cellular protein content and intracellular retention of Hoechst 33342 (by spectrofluorimetry: excitation and emission wavelengths were 355 and 460 nm, respectively), as described above.

Intracellular concentrations of reference substrates were initially expressed as fluorescence arbitrary units (for fluorescent dye substrates) or as moles (for radiolabeled substrates) and normalized to cellular protein content, determined by the Bradford method [44]. They were finally used for calculating percentage of transporter activity, according to the following equations [41, 45]:

$$\%\;\text{ABC}\;\text{transporter activity}=\frac{([{\text{Substrate}}_{\text{ref inh}}]\text{-}[{\text{Substrate}}_{\text{pyrethroid}}])\times 100}{\left([{\text{Substrate}}_{\text{ref inh}}]\text{-}[{\text{Substrate}}_{\text{control}}]\right)}$$ (1)

$$\%\;\text{SLC transporter activity}=\frac{([{\text{Substrate}}_{\text{pyrethroid}}]\text{-}[{\text{Substrat}}_{\text{ref inh}}])\times 100}{\left([{\text{Substrate}}_{\text{control}}]\text{-}[{\text{Substrate}}_{\text{ref inh}}]\right)}$$ (2)

with [Substrate_pyrethroid_] = cellular concentration of reference substrate in the presence of pyrethroid, [Substrate_ref inh_] = cellular concentration of reference substrate in the presence of reference inhibitor and [Substrate_control_] = cellular concentration of reference substrate in control cells not exposed to pyrethroid or reference inhibitor.

For OCT1-mediated transport inhibition by pyrethroids, data were also expressed as percentages of inhibition of OCT1 activity using the following equation:

%OCT1activity inhibition=100%-%OCT1activity(in the presence of pyrethroid) (3)

The initial tested concentration of pyrethroids was 100 μM. When a pyrethroid used a this concentration modulated transporter activity by more than 50%, the effects of various additional concentrations of the pyrethroid (from 0.1 to 300 μM) on transporter activity were analyzed, in order to determine half maximal inhibitory concentration (IC_50_) (for inhibitory effects of pyrethroids) or half maximal effective concentration (EC_50_) (for stimulatory effects of pyrethroids). Such concentrations were calculated using GraphPad Prism software (GraphPad Software, La Jolla, CA), through nonlinear regression based on the four parameter logistic function using the following equations:

For IC_50_:

$$\text{A}=\frac{100}{1+10^{\left(([\text{I}]\;\text{-}\;{\text{LogIC}}_{50})\text{x}\;\text{Hill slope}\right)}}$$ (4)

For EC_50_:

$$\text{A}=\frac{100}{1+10^{\left(([\text{I}]\;\text{-}\;{\text{LogEC}}_{50})\text{x}\;\text{Hill slope}\right)}}$$ (5)

where A is the percentage of transporter activity for a given concentration of pyrethroid determined as described in Eq (1) for ABC transporters and in Eq (2) for SLC transporters, [I] is the pyrethroid concentration in the medium, and Hill slope is a coefficient describing the steepness of the curve.

### Dopamine accumulation in OCT1-transfected HEK293 cells

HEK-MOCK and HEK-OCT1 cells were incubated with transport assay medium containing 11 nM 3,4-[Ring-2,5,6-^3^H]-dopamine, in the absence or presence of the reference OCT1 inhibitor verapamil or of pyrethroids for 5 min at 37°C in the transport assay medium described above. After washing twice with PBS, cells were lysed in distilled water and intracellular accumulation of dopamine was finally measured by scintillation counting.

### *Trans*-stimulation assays in OCT1-transfected HEK293 cells

*Trans*-stimulation assays were performed as previously described [46]. Briefly, OCT1-transfected HEK293 cells were first preloaded with 2 mM unlabeled TEA or 100 μM pyrethroid for 60 min at 37°c in the transport assay medium described above. After washing twice with PBS, cells were next re-incubated in transport assay medium containing 29 μM [1-^14^C]-TEA for 5 min at 37°C. After washing twice with PBS, cells were lysed in distilled water and intracellular accumulation of [1-^14^C]-TEA was then determined by scintillation counting.

### Molecular descriptor generation

Molecular descriptors were evaluated using the Dragon 6 software (Talete, Milano, Italy), that provides 4885 molecular descriptors divided into 29 blocks (See http://www.talete.mi.it/products/dragon_molecular_descriptor_list.pdf for a complete list of these descriptors). Pyrethroids, initially expressed in SMILES format, were converted to 3D format using the MarvinView software (ChemAxon, Budapest, Hungary) before processing by Dragon 6 to obtain molecular descriptors.

### Statistical analysis

Experimental data were expressed as means ± SEM of at least three independent experiments, each being usually performed in triplicate (for transport assays, one typical independent experiment therefore corresponded to 3 independent wells on the same multiwell plate). They were statistically analysis through the Student's *t*-test or analysis of variance (ANOVA) followed by a Dunnett's or a Newman-Keuls post-hoc test; the criterion of significance was *p* < 0.05. Correlation between molecular descriptor indexes and percentages of OCT1 activity inhibition by pyrethroids was initially done with Dragon 6 software through Pearson correlation, using a cut-off for Pearson’s correlation coefficient of r > 0.8 (positive correlation) or r < -0.8 (negative correlation), as recommended by the software user’s manual and as previously described [47]. *P*-values for Pearson correlations, as well as linear regressions for molecular descriptors exhibiting a high level of correlation with OCT1 activity inhibition (|r| > 0.92), were next determined using GraphPad Prism software after confirmation of normality of data distribution by D'Agostino and Pearson omnibus normality test. The statistical analysis of molecular descriptors belonging to the block of constitutional indices or to that of molecular properties and allowing to discriminate allethrin/tetramethrin from the other pyrethroids was performed using the unpaired Student’s *t*-test, which is applicable to very small sample sizes [48, 49].

## Results

### Effects of pyrethroids on ABC transporter activity

For investigating the effects of pyrethroids on ABC transporter activity, we used MCF7R, HuH-7 and HEK-BCRP cells. Such cells exhibited increased accumulation (MCF7R and HuH-7 cells) or retention (HEK-BCRP cells) of dyes substrates for P-gp (rhodamine 123), MRP2 and MRP2-like transporters (CF) or BCRP (Hoechst 33342) in response to reference inhibitors of the pumps (verapamil for P-gp, probenecid for MRP2 and fumitremorgin C for BCRP) (S2 Fig), thus demonstrating that P-gp, MRP2 and BCRP are fully functional in these cells, as previously described [45]. By contrast, verapamil failed to enhance rhodamine 123 accumulation in parental MCF7 cells (S2 Fig), which is fully consistent with the absence of detectable P-gp activity in these cells; in the same way, fumitremorgin C did not augment Hoechst 33342 retention in HEK-MOCK cells (data not shown).

**Fig 1:**
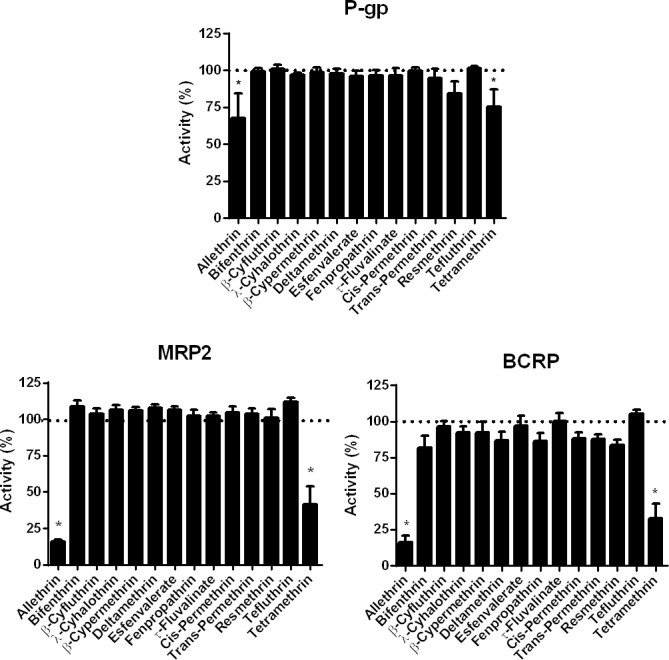
Effects of pyrethroids on ABC drug transporter activities. Activities of P-gp, MRP2 and BCRP were determined in ABC transporter-expressing cells (MCF7R cells for P-gp, HuH-7 cells for MRP2 and HEK-BCRP cells), in the absence or presence of reference inhibitors or various pyrethroids (each used at 100 μM), as described in Materials and Methods. Data are expressed as percentages of activities found in untreated control cells, arbitrarily set at 100% and indicated by dotted lines on graphs; they are the means ± SEM of three independent assays, each being performed in triplicate. *, p<0.05 when compared to untreated control cells.

Pyrethroids were routinely used at 100 μM for screening their potential effects towards drug transporter activity. This 100 μM concentration was retained because it was in the range of those previously used to interact with the pharmacological targets of pyrethroids, *i*.*e*., voltage-gated sodium channels, in cultured cells [50, 51], thus underlining its potential relevance for *in vitro* studying interactions of pyrethroids with membrane proteins. P-gp activity was found to be moderately, but significantly, inhibited (32.7% inhibition) by 100 μM allethrin (Fig 1), whereas MRP2 and BCRP activity were more potently inhibited (83.8% and 83.9% inhibition for MRP2 and BCRP, respectively) by the pyrethroid (Fig 1). For MRP2 inhibition, allethrin IC_50_ value was 48.2 μM (S3A Fig) whereas it was 42.5 μM for BCRP inhibition (S3B Fig). The pyrethroid tetramethrin used at 100 μM also notably decreased MRP2 activity (58.3% inhibition) and BCRP activity (67.1% inhibition), whereas P-gp activity was more weakly impaired (24.5% inhibition) (Fig 1); tetramethrin IC_50_ values were 65.5 μM (for MRP2 inhibition) (S3A Fig) and 72.5 μM (for BCRP inhibition) (S3B Fig). For P-gp, because 100 μM allethrin and 100 μM tetramethrin inhibited activity by less than 50%, IC_50_ values may be predicted to be higher than 100 μM. Other pyrethroids used at 100 μM did not significantly inhibit P-gp, MRP2 or BCRP activity (Fig 1).

### Effects of pyrethroids on organic anion SLC transporter activity

For investigating effects of pyrethroids on activity of organic anion SLC transporters, *i*.*e*., OATPs and OATs, we used OATP1B1-, OATP1B3-, OATP2B1-, OAT1- and OAT3-transfected cells. As shown in S4 Fig, these cells exhibited increased accumulation of reference substrates, *i*.*e*., E3S for OATP1B1 and OATP2B1 and fluorescein for OATP1B3, OAT1 and OAT3, when compared to OATP- or OAT-untransfected counterparts; moreover, the OATP inhibitor probenecid was able to reduce accumulation of reference substrates only in OATP- and OAT-transfected cells (S4 Fig). Taken together, these data indicated that OATPs and OATs were fully functional in OATP- and OAT-transfected cells.

**Fig 2:**
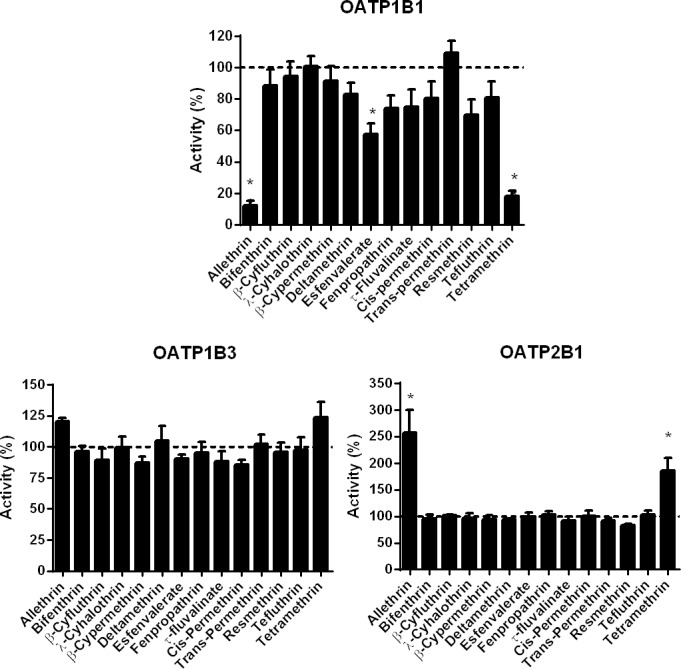
Effects of pyrethroids on OATP activities. Activities of OATP1B1, OATP1B3 and OATP2B1 were determined in OATP-transfected-cells in the absence or presence of reference inhibitors or various pyrethroids (each used at 100 μM), as described in Materials and Methods. Data are expressed as percentages of activities found in untreated control cells, arbitrarily set at 100% and indicated by dotted lines on graphs; they are the means ± SEM of three independent assays, each being performed in triplicate. *, p<0.05 when compared to untreated control cells.

As shown in Fig 2, allethrin and tetramethrin used at 100 μM, were found to markedly inhibit OATP1B1 activity, by more than 85%; corresponding IC_50_ values were 16.5 μM and 5.7 μM for allethrin and tetramethrin, respectively (S5A Fig). Other pyrethroids did not inhibit OATP1B1 activity or only moderately inhibited it (by less than 50%), without reaching statistical significance, except for esfenvalerate (Fig 2). None of pyrethroids was found to impair OATP1B3 activity (Fig 2). With respect to OATP2B1 activity, it was unexpectedly stimulated by allethrin (EC_50_ = 37.8 μM) and tetramethrin (EC_50_ = 10.1 μM) (Fig 2 and S5B Fig), whereas other pyrethroids had no significant effect (Fig 2).

**Fig 3:**
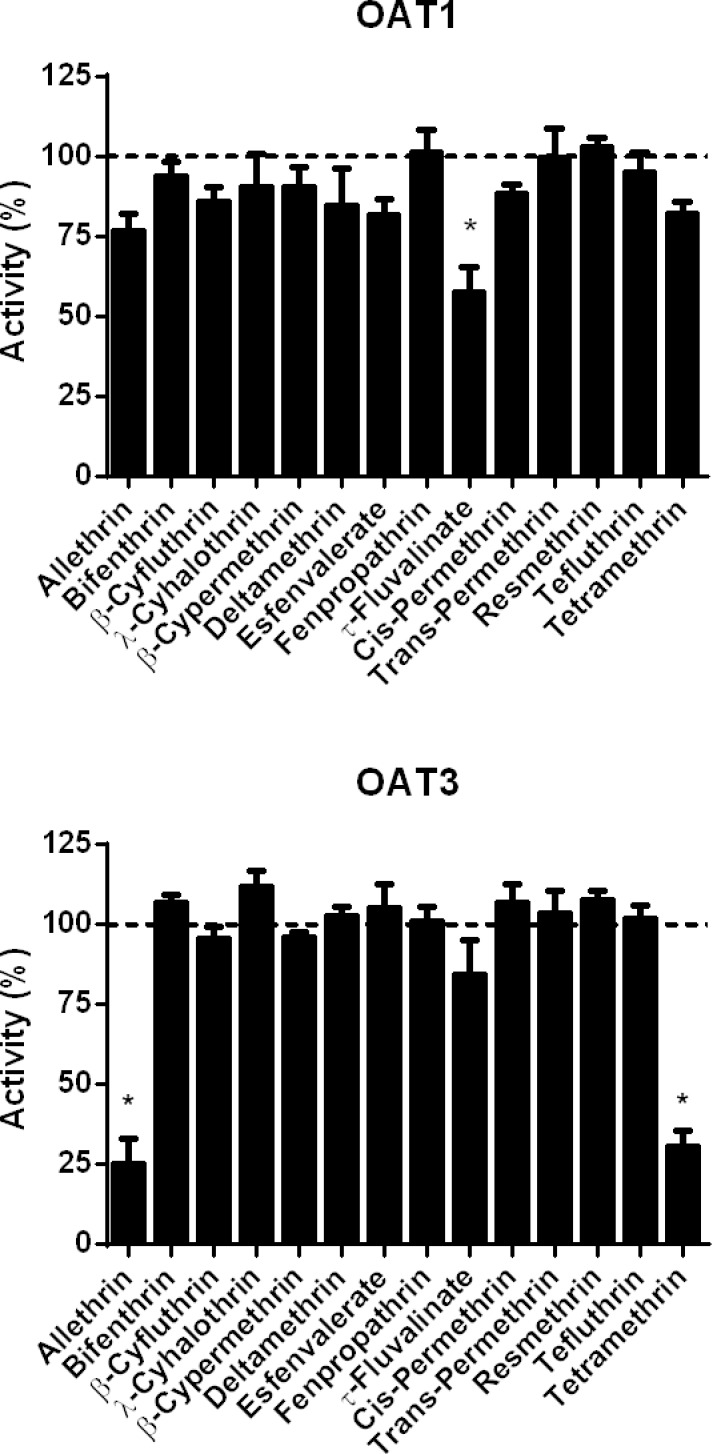
Effects of pyrethroids on OAT activities. Activities of OAT1 and OAT3 were determined in OAT-transfected HEK cells in the absence or presence of reference inhibitors or various pyrethroids (each used at 100 μM), as described in Materials and Methods. Data are expressed as percentages of activities found in untreated control cells, arbitrarily set at 100% and indicated by dotted lines on graphs; they are the means ± SEM of three independent assays, each being performed in triplicate. *, p<0.05 when compared to untreated control cells.

τ-fluvalinate, used at 100 μM, was the only pyrethroid that decreased OAT1 activity (Fig 3); the percentage of inhibition (42.8%) was however less than 50%, which precluded determination of IC_50_ value. τ-fluvalinate also inhibited OAT3 activity, but only in a marginal and non-significant manner (15.6% inhibition) (Fig 3). By contrast, OAT3 activity was more strongly and significantly inhibited by 100 μM allethrin (74.8% inhibition) and 100 μM tetramethrin (69.3% inhibition), whereas other pyrethroids failed to impair it (Fig 3); IC_50_ values for OAT3 inhibition were 69.4 μM (allethrin) and 77.6 μM (tetramethrin) (S5C Fig).

### Effects of pyrethroids on organic cation SLC transporter activity

For analyzing effects of pyrethroids on activity of organic cation SLC transporters, *i*.*e*., MATEs and OCTs, we used MATE1-, MATE2-K-, OCT1- and OCT2-transfected HEK293 cells. As shown in S6 Fig, these cells exhibited increased accumulation of reference substrates, *i*.*e*., TEA for MATE1 and MATE2-K, DAPI for OCT1 and rhodamine 123 for OCT2 when compared to HEK-MOCK cells; moreover, reference inhibitors (verapamil for MATEs and OCT1 and amitriptyline for OCT2) reduced accumulation of reference substrates in MATE- and OCT-transfected cells, but not in HEK-MOCK cells (S6 Fig). Taken together, these data indicated that MATEs and OCTs were fully functional in MATE- and OCT-transfected cells.

**Fig 4:**
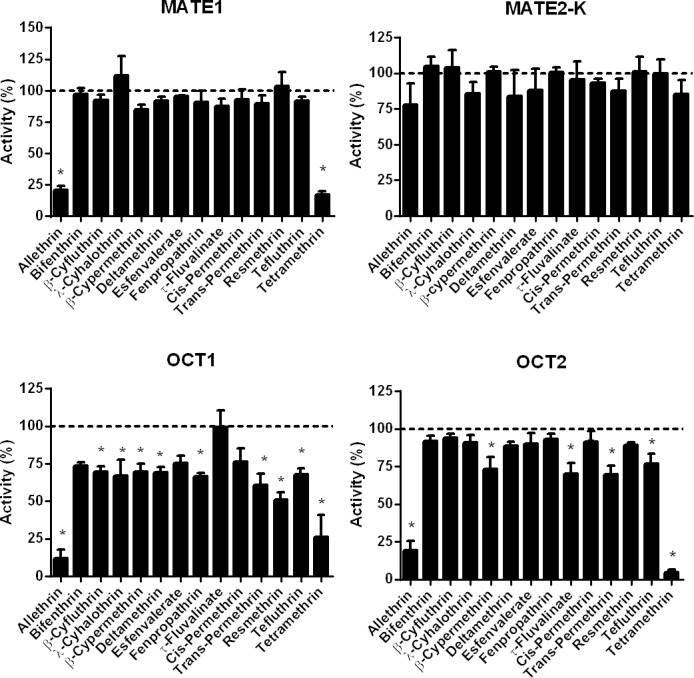
Effects of pyrethroids on MATE and OCT activities. Activities of MATE1, MATE2-K, OCT1 and OCT2 were determined in MATE- or OCT-transfected HEK cells, in the absence or presence of reference inhibitors or various pyrethroids (each used at 100 μM), as described in Materials and Methods. Data are expressed as percentages of activities found in untreated control cells, arbitrarily set at 100% and indicated by dotted lines on graphs; they are the means ± SEM of three independent assays, each being usually performed in triplicate. *, p<0.05 when compared to untreated control cells.

As shown in Fig 4, allethrin and tetramethrin, used at 100 μM, markedly inhibited MATE1 activity, by more than 79%; corresponding IC_50_ values were 50.2 μM and 47.5 μM for allethrin and tetramethrin, respectively (S7A Fig). Other pyrethroids did not significantly inhibit MATE1 activity (Fig 4). None of pyrethroids was found to impair MATE2-K activity (Fig 4).

With respect to OCT1 activity, it was markedly inhibited by 100 μM allethrin (87.9% inhibition) and 100 μM tetramethrin (73.8% inhibition); corresponding IC_50_ values, *i*.*e*., 2.6 μM (allethrin) and 4.9 μM (tetramethrin), were rather low (S7B Fig). Pyrethroids such as β-cyfluthrin, λ-cyhalothrin, β-cypermethrin, deltamethrin, fenpropathrin, *trans*-permethrin, resmethrin and tefluthrin also significantly inhibited OCT1 activity, but in a weaker manner; OCT1 inhibition thus ranged from 30.1% (β-cypermethrin) to 48.8% (resmethrin) (Fig 4). By contrast, bifenthrin, *cis*-permethrin, esfenvalerate and τ-fluvalinate failed to significantly alter OCT1 activity (Fig 4). Allethrin and tetramethrin were finally also shown to markedly inhibit OCT2 activity (Fig 4); IC_50_ values were 42.6 μM (allethrin) and 11.2 μM (tetramethrin) (S7C Fig). OCT2 activity was also counteracted by β-cypermethrin, τ-fluvalinate, *trans*-permethrin and tefluthrin, but in a weaker manner with a percentage of inhibition around 25% (Fig 4). Other pyrethroids failed to impair OCT2-mediated transport (Fig 4).

### Characterization of pyrethroids-mediated OCT1 inhibition

We next focused on pyrethroids-mediated OCT1 inhibition. Indeed, OCT1 can be considered as the drug transporter most impacted by pyrethroids because it was the transporter most sensitive to allethrin and tetramethrin according to IC_50_ values (S7B Fig) and it was also the transporter significantly inhibited by the largest number of pyrethroids, as 10/14 pyrethroids significantly impaired its activity (Fig 4).

**Fig 5:**
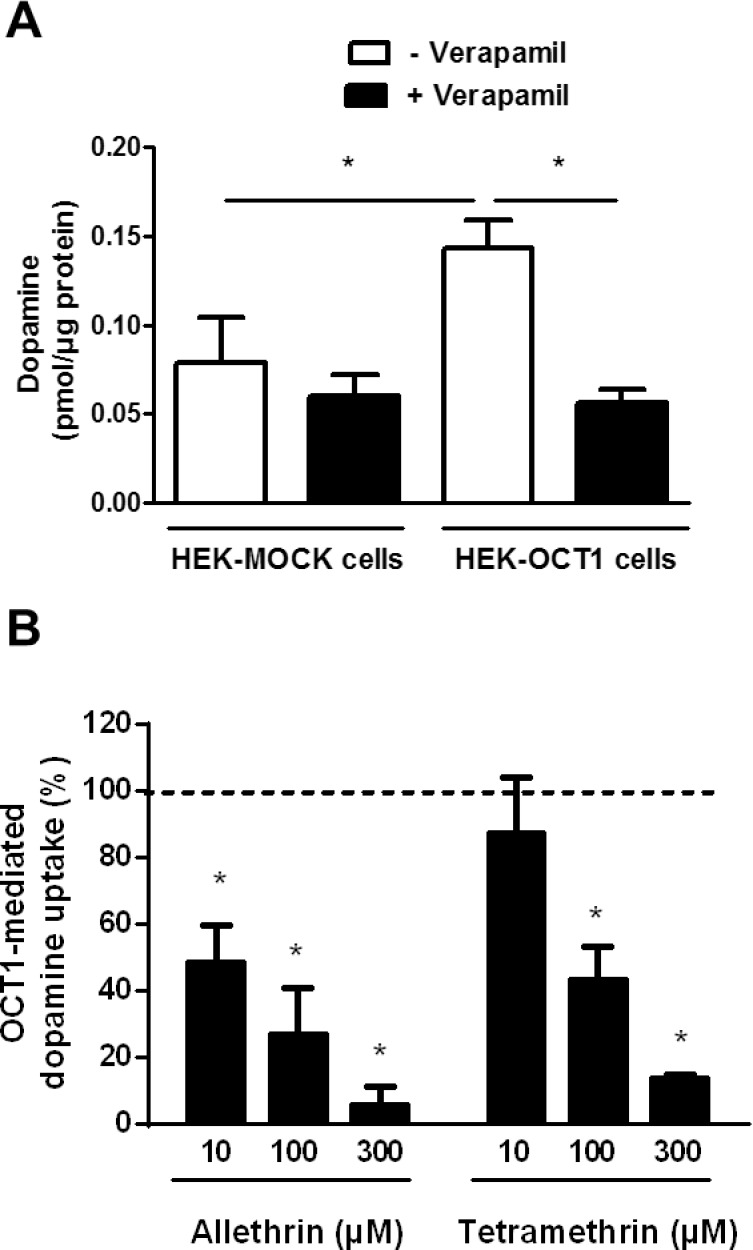
Effects of allethrin and tetramethrin on OCT1-mediated transport of dopamine. (A) HEK-OCT1 and HEK-MOCK cells were incubated with 11 nM 3,4-[Ring-2,5,6-^3^H]-dopamine in the absence or presence of the reference OCT1 inhibitor verapamil (100 μM) for 5 min at 37°C. Intracellular accumulation of 3,4-[Ring-2,5,6-^3^H]-dopamine was then determined by scintillation counting. Data are expressed as pmol/μg protein and are the means ± SEM of three independent experiments, each being performed in triplicate. *, p<0.05. (B) HEK-OCT1 cells were incubated with 11 nM 3,4-[Ring-2,5,6-^3^H]-dopamine in the absence or presence of various concentrations (10, 100 and 300 μM) of allethrin or tetramethrin. Intracellular accumulation of 3,4-[Ring-2,5,6-^3^H]-dopamine was then determined by scintillation counting. Data are expressed as percentages of OCT1-mediated transport of dopamine in control pyrethroid-unexposed cells, determined by subtracting accumulation of dopamine in HEK-MOCK cells from that in HEK-OCT1 cells in the absence of pyrethroids, arbitrarily set at 100% and indicated by a dotted line on the graph. They are the means ± SEM of at least three independent assays, each being performed in triplicate. *, p<0.05 when compared to untreated control cells.

**Fig 6:**
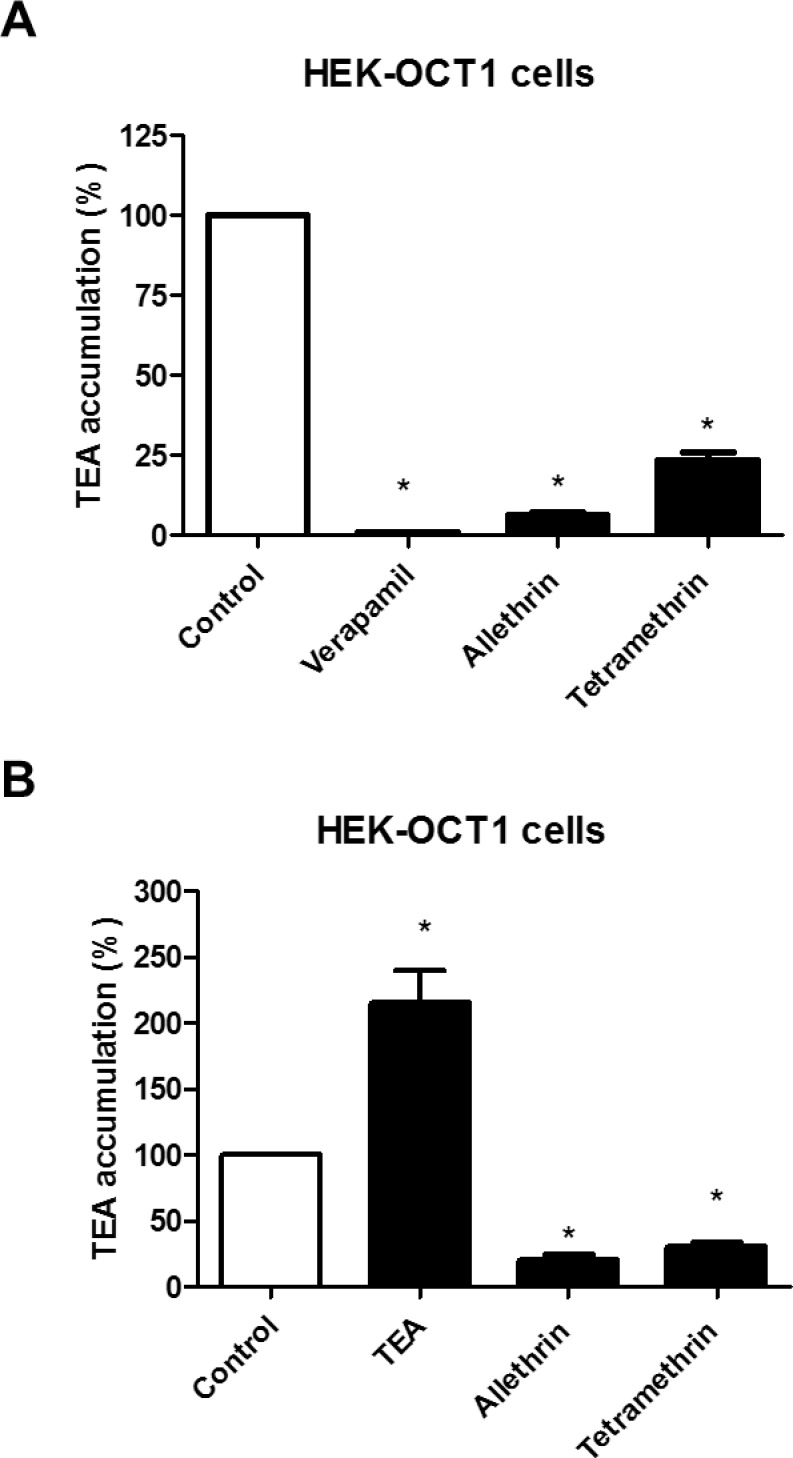
*Cis*- and *trans*-inhibitory effects of allethrin and tetramethrin towards OCT1-mediated transport of TEA. (A) HEK-OCT1 cells were incubated with 29 μM [1-^14^C]-TEA for 5 min at 37°C in the absence (control) or presence of 100 μM verapamil, 100 μM allethrin or 100 μM tetramethrin. Intracellular accumulation of TEA was then determined by scintillation counting. Data are expressed as percentages of TEA accumulation in control cells and are the means ± SEM of three independent experiments, each being performed in triplicate. *, p<0.05 when compared to control cells. (B) HEK-OCT1 cells were first incubated for 60 min at 37°c in the absence (control) or presence of 2 mM unlabeled TEA, 100 μM allethrin or 100 μM tetramethrin. After washing, cells were next re-incubated with 29 μM [1-^14^C]-TEA for 5 min at 37°C. Intracellular accumulation of [1-^14^C]-TEA was then determined by scintillation counting. Data are expressed as percentages of TEA accumulation in control cells and are the means ± SEM of three independent experiments, each being performed in triplicate. *, p<0.05 when compared to control cells.

We first determined whether allethrin and tetramethrin can inhibit OCT1-mediated transport of endogenous substrates such as dopamine. HEK-OCT1 cells exhibited enhanced verapamil-inhibitable accumulation of dopamine comparatively to HEK-MOCK cells (Fig 5A), thus fully confirming that OCT1 handles dopamine [52]. Allethrin used at 10, 100 or 300 μM was found to significantly inhibit this OCT1-mediated transport of dopamine (Fig 5B); tetramethrin also decreased it, but only when used at 100 or 300 μM (Fig 5B). Allethrin and tetramethrin used at 100 μM were next shown to markedly *cis*-inhibit accumulation of the reference OCT1 substrate TEA in HEK-OCT1 cells (Fig 6A). Finally, we investigated whether allethrin and tetramethrin can *trans*-stimulate TEA uptake, which may constitute an argument in favor of the transport of the two pyrethroids by OCT1 [46]. Pre-loading with allethrin and tetramethrin however resulted in *trans*-inhibition, and not *trans*-stimulation, of radiolabeled TEA uptake (Fig 6B). By contrast, pre-loading with unlabeled TEA led to a *trans*-stimulation of radiolabeled TEA uptake (Fig 6B), as expected for an OCT1 substrate like TEA [46].

In order to identify the specific physico-chemical properties associated to OCT1 inhibition by pyrethroids, molecular descriptors, including 0D-constitutional, 1D-structural, 2D-topological and 3D-geometrical descriptors, were determined using Dragon 6 software. Putative correlations with OCT1 activity inhibition were next analyzed using Pearson’s correlation test. Using a cutoff of |r| > 0.8, 602 molecular descriptors were found to be correlated with inhibition of OCT1 activity; the correlation was positive (r > 0.8) or negative (r < -0.8) for 93 and 509 descriptors, respectively (Table 1). These molecular descriptors associated with OCT1 inhibition belong to different blocks of descriptors, especially those of 2D-matrix based descriptors (n = 350), of edge adjacency indices (n = 85), of topological indices (n = 26), of 3D-MoRSE descriptors (n = 24) and of walk and path counts (n = 20) (Table 1). A complete list of these descriptors is given in S1 File. Notably, lipophilicity, evaluated through octanol-water partition coefficients (LogP) calculated using Moriguchi LogP model (MLOGP), was negatively correlated to OCT1 inhibition, whereas no correlation was found for molecular weight (MW) or number of hydrogen bond acceptors (nHAcc) and donors (nHDon) (S1 File). When a more stringent cut-off value of |r| > 0.92 was applied, 9 molecular descriptors were found to be correlated with OCT1 activity inhibition (S4 Table); linear regression analysis next confirmed a highly significant linear relation between the index values of these descriptors and the percentages of OCT1 activity inhibition (S8 Fig). Such molecular descriptors exclusively belong to the category of 2D topological indexes, notably to walk and path counts (S4 Table).

**Table 1:** Repartition of molecular descriptors correlated with OCT1 activity inhibition by pyrethroids (|r|>0.80).

Dragon ID Block	Block category	Block description	Number of descriptors	Number of correlated descriptors		Percentage of Correlated descriptors in each block	Percentage of total correlated descriptors
				Positive Correlation (r > 0.8)	Negative Correlation (r < -0.8)		
**1**	0D-descriptors	Constitutional descriptors	43	2	7	20.9	1.5
**2**	0D-descriptors	Ring descriptors	32	1	4	15.6	0.8
**3**	2D-descriptors	Topological indices	75	1	25	34.7	4.3
**4**	2D-descriptors	Walk and path counts	46	0	20	43.5	3.3
**5**	2D-descriptors	Connectivity indices	37	0	7	18.9	1.2
**6**	2D-descriptors	Information indices	48	0	9	18.8	1.5
**7**	2D-descriptors	2D matrix-based descriptors	550	36	314	63.6	58.1
**8**	2D-descriptors	2D autocorrelations	213	4	3	3.3	1.2
**9**	2D-descriptors	Burden eigenvalues	96	0	6	6.3	1.0
**10**	2D-descriptors	P_VSA-like descriptors	45	1	1	4.4	0.3
**11**	2D-descriptors	ETA indices	23	1	8	39.1	1.5
**12**	2D-descriptors	Edge adjacency indices	324	8	77	26.2	14.1
**13**	3D-descriptors	Geometrical descriptors	38	0	5	13.2	0.8
**14**	3D-descriptors	3D matrix-based descriptors	90	3	2	5.6	0.8
**15**	3D-descriptors	3D autocorrelations	80	1	0	1.3	0.2
**16**	3D-descriptors	RDF descriptors	210	0	2	1.0	0.3
**17**	3D-descriptors	3D-MoRSE descriptors	224	20	4	10.7	4.0
**18**	3D-descriptors	WHIM descriptors	114	0	0	0.0	0.0
**19**	3D-descriptors	GETAWAY descriptors	273	0	9	3.3	1.5
**20**	3D-descriptors	Randic molecular profiles	41	1	0	2.4	0.2
**21**	0D-descriptors	Functional group counts	154	2	2	2.6	0.7
**22**	1D-descriptors	Atom-centred fragments	115	3	0	2.6	0.5
**23**	1D-descriptors	Atom-type E-state indices	170	1	1	1.2	0.3
**24**	2D-descriptors	CATS 2D	150	0	0	0.0	0.0
**25**	2D-descriptors	2D Atom Pairs	1596	3	0	0.2	0.5
**26**	3D-descriptors	3D Atom Pairs	36	0	0	0.0	0.0
**27**	others	Charge descriptors	15	0	0	0.0	0.0
**28**	others	Molecular properties	20	0	3	15	0.5
**29**	others	Drug-like indices	27	5	0	18.5	0.9

### Determination of basic molecular descriptors discriminating multi-transporter-interacting pyrethroids (allethrin/tetramethrin) from other pyrethroids

**Fig 7:**
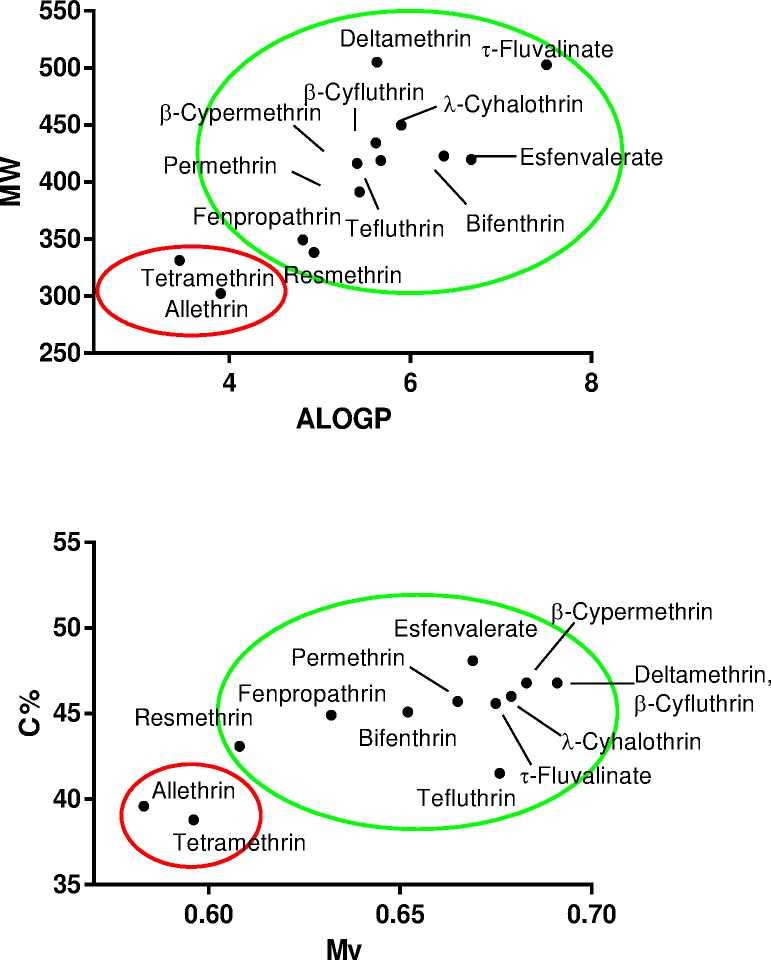
Graphical pairwise representation of some molecular descriptors allowing to discriminate allethrin/tetramethrin from other pyrethoids. Allethrin/tetramethrin are graphically included in a red circle, whereas other pyrethoids are included in a green circle. MW, molecular weight; ALOGP, Ghose-Crippen-Viswanadhan octanol-water partition coefficient; Mv, mean atomic van der Waals volume (scaled on Carbon atom); C%, percentage of C atoms.

**Fig 8:**
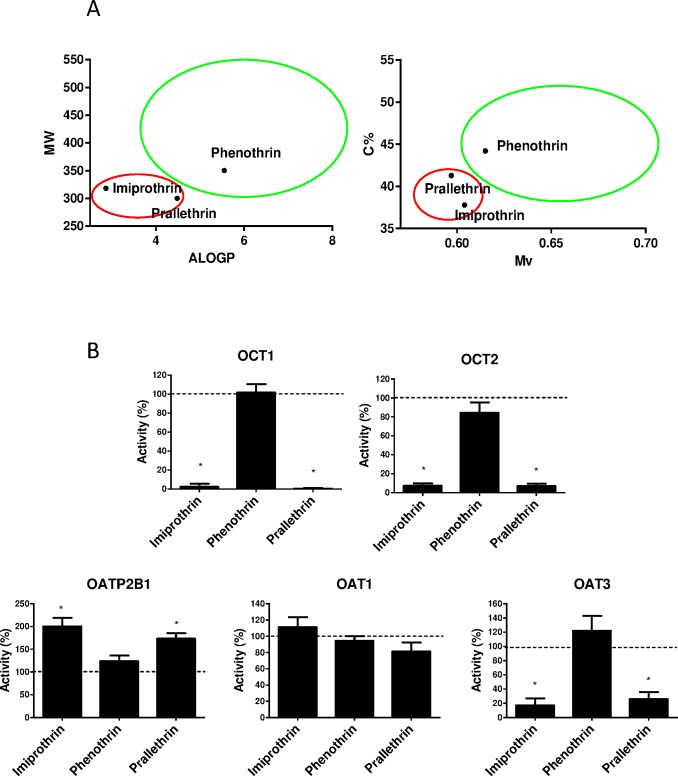
Effects of imiprothrin, phenothrin and prallethrin on OCT1, OCT2, OATP2B1, OAT1 and OAT3 activities. (A) Imiprothrin, phenothrin and prallethrin were projected on graphs MW (molecular weight) versus ALOGP (Ghose-Crippen-Viswanadhan octanol-water partition coefficient) (Right panel) and C % (percentage of C atoms) versus Mv (mean atomic van der Waals volume, scaled on Carbon atom) (Left panel). Red and green circles were placed exactly as defined in Fig 7 and putatively discriminated pyrethroids interacting with drug transporters (red circle) from those that do not it (green circle). (B) Activities of OCT1, OCT2, OATP2B1, OAT1 and OAT3 were determined in transporter-transfected HEK cells in the absence or presence of reference inhibitors or of imiprothrin, phenothrin or prallethrin (each used at 100 μM), as described in Materials and Methods. Data are expressed as percentages of activities found in untreated control cells, arbitrarily set at 100% and indicated by dotted lines on graphs; they are the means ± SEM of at least three independent assays, each being usually performed in triplicate. *, p<0.05 when compared to untreated control cells.

Allethrin and tetramethrin exhibit a rather large, but specific, profile of transporter inhibition, because they markedly decreased activity of various transporters, including MRP2, BCRP, OATP1B1, OAT3, MATE1, OCT1 and OCT2, whereas other pyrethroids concomitantly exerted no, or only moderate or marginal, inhibitory effects. In addition, allethrin and tetramethrin, unlike other pyrethroids, stimulated OATP2B1 activity (Fig 2). Molecular features restricted to allethrin and tetramethrin may therefore be hypothesized to be involved in the regulations of drug transporter activities caused by the two pyrethroids. In order to identify such physical-chemical parameters, we compared constitutional indices and molecular properties from allethrin and tetramethrin with those of other pyrethroids, because important descriptors for transporter inhibition are usually comprised among these basic descriptors [53]. As shown in Table 2, values for 19 descriptors, *i*.*e*., 15 constitutional indices and 4 molecular properties, were found to significantly differ between allethrin/tetramethrin and other pyrethroids. These discriminating molecular descriptors notably correspond to molecular weight (MW), van der Waals volume (Mv), polarizability (Mp), rotatable bond fraction (RBF), percentage of C atoms (C%) and lipophilicity/LogP, calculated using either Moriguchi (MLOGP) or Ghose-Crippen-Viswanadhan (ALOGP) LogP models. Allethrin/tetramethrin thus exhibit reduced molecular weight, van der Waals volume, polarizability, percentage of C atoms and lipophilicity when compared to other pyrethroids (Table 2). Interestingly, when some of these descriptors were combined pairwise, they allowed to easily graphically discriminate allethrin/tetramethrin from other pyrethroids (Fig 7). Applying this graphically discrimination based on MW versus ALOGP or on C% versus Mv to three type I pyrethroids not previously analyzed in the present study, *i*.*e*., imiprothrin, prallethrin and phenothrin, allowed to predict that imiprothrin and prallethrin, unlike phenothrin, may interact with drug transporter activities (Fig 8A). This prediction was next validated through demonstrating that imiprothrin and prallethrin, but not phenothrin, inhibited OCT1, OCT2 and OAT3 activities and cis-stimulated that of OATP2B1 when used at 100 μM (Fig 8B); imiprothrin and prallethrin, like allethrin and tetramethrin (Fig 3), however failed to alter OAT1 activity (Fig 8B).

**Table 2:** Molecular descriptors discriminating allethrin/tetramethrin from other pyrethroids.

Molecular descriptor			Index mean		*p*-value^a^
Name	Description	Block	Allethrin- Tetramethrin	Other pyrethroids	
MW	molecular weight	CI^b^	317.0	422.7	0.0198
AMW	average molecular weight	CI	6.5	8.7	0.0321
Mv	mean atomic van der Waals volume (scaled on Carbon atom)	CI	0.590	0.666	0.0021
Mp	mean atomic polarizability (scaled on Carbon atom)	CI	0.631	0.691	0.135
nSK	number of non-H atoms	CI	23.0	28.5	0.0221
nBO	number of non-H bonds	CI	24.5	30.5	0.0223
SCBO	sum of conventional bond orders (H-depleted)	CI	29.5	39.7	0.0123
RBF	rotatable bond fraction	CI	0.110	0.136	0.0348
nH	number of Hydrogen atoms	CI	25.50	20.36	0.0478
H%	percentage of H atoms	CI	52.60	41.51	0.008
C%	percentage of C atoms	CI	39.20	45.49	0.0008
O%	percentage of O atoms	CI	7.25	5.78	0.0488
nCsp3	number of sp3 hybridized Carbon atoms	CI	11.50	6.82	0.0004
nCsp2	number of sp2 hybridized Carbon atoms	CI	7.50	14.82	0.004
AMR	Ghose-Crippen molar refractivity	MP^c^	89.45	107.59	0.0447
MLOGP	Moriguchi octanol-water partition coeff.(logP)	MP	3.478	4.497	0.0017
ALOGP	Ghose-Crippen octanol-water partition coeff.(logP)	MP	3.676	5.814	0.0034
ALOGP2	squared Ghose-Crippen octanol-water partition coeff. (logP^2)	MP	13.567	34.581	0.0131
PDI	packing density index	MP	0.900	0.928	0.0196

^a^ given by Student’s *t* test.

^b^ constitutional indice.

^c^ molecular property.

## Discussion

Previous studies have shown that some pesticides, including pyrethroids, may interact with drug transporters [26, 27, 54, 55]. The data reported in the present study fully support this hypothesis through demonstrating that the type I pyrethroids allethrin and tetramethrin inhibited various ABC and SLC drug transporters (See Table 3 for a summary of drug transporter activity regulation by these two pyrethroids). The type I pyrethroids imiprothrin and prallethrin were additionally shown to interact with OCT1, OCT2, OAT3 and OATP2B1 activities. By contrast, other type I or type II pyrethroids, did not, or only rather very moderately or marginally, impair drug transporter activities.

**Table 3:** Summary of allethrin and tetramethrin effects towards drug transporters.

Drug transporter		Allethrin	Tetramethrin
ABC transporter	P-gp	Inhibition (IC_50_>100 μM)	Inhibition (IC_50_>100 μM)
	MRP2	Inhibition (IC_50_ = 48.2 μM)	Inhibition (IC_50_ = 65.5 μM)
	BCRP	Inhibition (IC_50_ = 42.5 μM)	Inhibition (IC_50_ = 72.5 μM)
SLC transporter	OATP1B1	Inhibition (IC_50_ = 16.5 μM)	Inhibition (IC_50_ = 5.7 μM)
	OATP1B3	No effect (up to 100 μM)	No effect (up to 100 μM)
	OATP2B1	Stimulation (EC_50_ = 37.8 μM)	Stimulation (EC_50_ = 10.1 μM)
	OAT1	No effect (up to 100 μM)	No effect (up to 100 μM)
	OAT3	Inhibition (IC_50_ = 69.4 μM)	Inhibition (IC_50_ = 77.6 μM)
	MATE1	Inhibition (IC_50_ = 50.2 μM)	Inhibition (IC_50_ = 47.5 μM)
	MATE2-K	No effect (up to 100 μM)	No effect (up to 100 μM)
	OCT1	Inhibition (IC_50_ = 2.6 μM)	Inhibition (IC_50_ = 4.9 μM)
	OCT2	Inhibition (IC_50_ = 42.6 μM)	Inhibition (IC_50_ = 11.2 μM)

Allethrin and tetramethrin significantly inhibited activities of the drug efflux pumps P-gp, MRP2 and BCRP and those of the SLC transporters OATP1B1, OAT3, MATE1, OCT1 and OCT2. The potency of inhibition nevertheless depends on transporters (Table 3). According to IC_50_ values, that constitute the main parameter to be considered for drug transporter inhibition, notably under a regulatory point of view [22], transporter ranking (from the most inhibited to the less inhibited transporter) was OCT1>OATP1B1>BCRP>OCT2>MRP2>MATE1>OAT3>P-gp for allethrin and OCT1>OATP1B1>OCT2>MATE1>MRP2>BCRP>OAT3>P-gp for tetramethrin. The two pyrethroids also interacted with OATP2B1 activity, which was *cis*-stimulated. Only OATP1B3, OAT1 and MATE2-K activities were not altered by the two pyrethroids. The fact that allethrin and tetramethrin failed to interact with these drug transporters, associated to the stimulation of OATP2B1 and to the differential sensitivities of inhibited transporters, discards any general and non-discriminating inhibitory effect of allethrin and tetramethrin towards membrane transporter activities. In addition, IC_50_ for transporter inhibition by allethrin and tetramethrin range from 2.6 μM to 77.6 μM, and such values are generally much higher than pyrethroid concentrations (around 0.5–2 μM) usually required for interacting with sodium channels in primary cultured mammalian cells [56], making unlikely that drug transporter inhibition by pyrethroids may be due to alteration of sodium channel activity. This conclusion is also fully supported by the fact that the majority of pyrethroids, excepted allethrin, tetramethrin, imiprothrin and prallethrin, failed to inhibit transporters in a major and notable way, even if they are all presumed to efficiently alter sodium channel activity [35]. Interactions of some pyrethroids such as allethrin and tetramethrin with various drug transporters can rather be considered as specific, most likely reflecting direct and transporter-dependent interactions of these two pyrethroids with substrate and/or regulatory binding sites on drug transporters, as classically thought for drug transporter inhibitors [57]. In this context, it can be speculated that binding of allethrin and tetramethrin to targeted transporters reflects physico-chemical properties specific to these two pyrethroids. Such properties are likely unrelated to those underlying activity of allethrin and tetramethrin towards sodium channels. Indeed, the sodium channels-based insecticidal activity of allethrin and tetramethrin is strictly dependent upon the entire stereospecific structure of these insecticides [9, 34], as for other pyrethroids inactive or poorly active on transporters. It is consequently restricted to some stereoisomers and did not rely on a specific substructure reactive entity or molecular moiety that could be identified as the toxophore conferring pyrethroid-like insecticidal activity [35]. The inhibitory effects against transporter activities of allethrin and tetramethrin can therefore not been specifically ascribed to the pyrethroid isomers within these mixtures that are insecticides and mammalian neurotoxicants. Further studies are therefore likely required to investigate the exact contribution of the various tetramethrin and allethrin stereoisomers not implicated in primary insecticidal and neurotoxic effects to drug transporter inhibition, knowing that the effects of drug transporter inhibitors may exhibit stereoselectivity [58].

Physicochemical parameters involved in drug transporter inhibitory effects exerted by allethrin and tetramethrin likely include constitutional indices, such as molecular weight and polarizability, and molecular properties, such as hydrophobicity (LogP), which were found to significantly discriminate allethrin/tetramethrin from other pyrethroids. Importantly, these basic descriptors have already been shown to be crucial for inhibition of the transporters targeted by the two pyrethroids. Lipophilicity/LogP, molecular weight and/or molecular polarizability thus constitute important parameters to consider for inhibiting MRP2, BCRP, OATP1B1, OAT3, MATE1, OCT1 or OCT2 [59–65]. Interestingly, pairwise association of some of these basic descriptors allow to easily and graphically differentiate allethrin/tetramethrin from other pyrethroids not, or only poorly interacting with drug transporters. Such a graphical discrimination was moreover used to successfully predict whether pyrethroids such as imiprothrin, phenothrin and prallethrin may interact with activity of transporters like OCT1, OCT2, OATP2B1, OAT1 and OAT3. This suggests that taking into consideration various descriptors together, including not only basic descriptors but also 2D- and 3D-molecular descriptors, would help to precisely and more extensively characterize the molecular basis of the specific interactions of some pyrethroids with drug transporters. In particular, this may lead to a better knowledge of the molecular features involved in *cis*-stimulation of OATP2B1 by pyrethroids like allethrin and tetramethrin. *Cis*-stimulation of OATP transporters has already been reported for other chemicals [60]. Similarly, other transporters like MRP2, OAT1, OAT3 and MATE1 are also subjected to *cis*-stimulation by some chemicals [61, 62, 66]. The molecular mechanisms that underlie such *cis*-stimulatory effects remain however yet poorly characterized.

Among transporters targeted by allethrin and tetramethrin, OCT1 is likely a major one. Indeed, allethrin and tetramethrin inhibited OCT1-mediated transport of DAPI with IC_50_ in the 2–5 μM range. Moreover, OCT1-mediated transport of the dye was similarly markedly inhibited by 100 μM imiprothrin and prallethrin, whereas other pyrethoids also impacted OCT1 activity, although in a weaker manner. Allethrin and tetramethrin also blocked OCT1-mediated transport of the reference OCT1 substrate TEA, as well as that of the endogenous substrate dopamine. Inhibition of OCT1-related transport of dopamine by the two pyrethroids was however rather less marked than that of DAPI; indeed, allethrin used at 10 μM reduced OCT1-mediated DAPI and dopamine accumulation by 73.7% and 51.6%, respectively, whereas 10 μM tetramethrin decreased DAPI uptake by 66.4%, but failed to significantly alter that of dopamine (S7B Fig and Fig 5). This likely illustrates the fact that inhibitory profiles for a transporter can vary according to the nature of the substrate and of drug binding sites, as already established for various transporters, including organic cation transporters [67–69]. The mechanism by which pyrethroids like allethrin and tetramethrin down-regulate OCT1 activity remain to be determined. Because the two pyrethroids failed to *trans*-stimulate OCT1 activity, but rather *trans*-inhibited it, they are likely not substrate for OCT1. This may suggest that they do not act through a competitive mechanism toward the transport drug binding site on OCT1. High lipophilicity has been previously shown to be positively correlated to OCT1 inhibition, whereas a high molecular dipole moment and many hydrogen bonds were negatively correlated and molecular weight was not correlated [63]. In agreement with these data, we have found that molecular weight was also not correlated with OCT1 inhibition by pyrethroids. By contrast, LogP was negatively correlated for pyrethroids, whereas the number of hydrogen bonds as well as those of hydrogen bond acceptors and donors were not correlated. The reason for these discrepancies between our study and that of Ahlin et al. [63] is unclear, but could be linked to the small number and the relative structural homology of pyrethroids analyzed in the present study (n = 14) when compared to the larger number of structurally-diverse chemicals (n = 191) previously investigated [63]. Moreover, all the analyzed pyrethroids are rather lipophilic and non-charged compounds. Nevertheless, a great number of molecular descriptors, notably 2D-matrix based descriptors, has been found to be correlated to OCT1 activity inhibition by pyrethroids; some of them, notably some walk and path counts, were moreover demonstrated to exhibit a highly significant linear relationship with the percentage of OCT1 activity inhibition. Beyond pyrethroids, further studies are likely required to evaluate the relevance of these molecular descriptors for predicting OCT1 inhibition by diverse structurally-unrelated chemicals.

The putative relevance of the transporter inhibitions described *in vitro* in the present study to human exposure to environmental pyrethroids is probably a key-point that has to be considered. In this context, it is first noteworthy that only some of the pyrethroids tested, *i*.*e*., allethrin, tetramethrin, imiprothrin and prallethrin, exhibit significant inhibitory effects towards transporters; this indicates that such effects on drug transporters are likely idiosyncratic off-target effects of a limited number of compounds rather than representative of effects relevant to the toxicology of pyrethroids as a class. Moreover, blood concentrations of pyrethroids are very low or not detected in human subjects environmentally exposed to these pesticides (<5μg/L, *i*.*e*., <12.8 nM for cyfluthrin, cypermethrin, deltamethrin or permethrin [70]). This makes very unlikely that pyrethroid concentrations required to inhibit drug transporters, which range from 2.6 μM to 77.6 μM for allethrin/tetramethrin according to IC_50_ values, and are therefore much higher than those targeting ion channels [56], may be reached *in vivo*. Moreover, following systemic absorption, pyrethroids are rapidly and extensively metabolized through oxidation, ester hydrolysis and conjugation, which precludes their accumulation in any specific tissues or organs [71]. Transporter inhibition in response to environmental exposure to pyrethroids such as allethrin and tetramethrin is therefore unlikely to occur at first view, which discards any drug-drug interactions due to drug transport impairment or any alteration of endogenous substrate transport by environmental pyrethroids. Putative consideration of the inhibitory effects of pyrethroids like allethrin and tetramethrin towards drug transporters under a regulatory point of view for pyrethroid risk assessment can therefore be excluded. It should be however kept in mind that humans are likely exposed not only to a single pyrethroid, but to other pesticides and xenobiotics, that may also interact with drug transporters [55], as recently demonstrated for notably organochlorine pesticides [43], polychlorinated biphenyls [72], diesel exhaust particle components [41] and perfluorinated surfactants [73]. Plasma levels of pyrethroids, especially allethrin and tetramethrin, associated with those of other pollutants, may therefore be sufficient to contribute to synergic or additive inhibitory effects towards drug transporters, as already described for pesticide combinations [74]. In addition, concentrations of pyrethroids may be much higher in the gastro-intestinal tract (before first-pass metabolism) than in plasma, and activities of drug transporters notably expressed at the intestinal level, especially P-gp, MRP2, BCRP, OATP2B1 and OCT1 [22, 75], may therefore be hypothesized to be impacted by ingested pyrethroids. Moreover, characterizing concentration-dependence of transporter activity inhibition by allethrin and tetramethrin in the present study was based on IC_50_ values; because IC_50_ values rely on substrate concentrations [76], the relevance of potential transporter inhibition has likely to be challenged according to the nature and *in vivo* concentrations of putative substrates. Finally, elevated concentrations of pyrethroids are likely to occur during acute poisoning [77] and, in this case, inhibition of transport of endogenous substrates like dopamine by allethrin or tetramethrin may contribute to the toxicity of these pesticides.

Another points that should be considered are the putative handling of pyrethroids by drug transporters, notably by intestinal transporters which in case of food and drinking water exposure would first be encountered [29], and the consequences for toxicokinetics of these insecticides. In this context, it is noteworthy that whether pyrethroids or their metabolites can be substrates for drug transporters remains to be determined. As already discussed above, transport of allethrin and tetramethrin by OCT1 is however unlikely to occur, as demonstrated by the failure of these two chemicals to *trans*-stimulate OCT1 activity. In the same way, deltamethrin has been recently shown to be not effluxed by P-gp [78], which agrees with the fact that this pyrethroid did not inhibit this ABC efflux pump in the present study and also failed to alter P-gp ATPase activity [28]. Conflicting results have however been reported with respect to interactions of pyrethroids with P-gp. Cypermethrin, esfenvalerate, fluvalinate and permethrin have thus been shown to inhibit P-gp activity or ATPase activity [26, 27], but they did not alter P-gp activity in the present study. Similarly, permethrin and resmethrin inhibited BCRP ATPase activity [28], without inhibiting BCRP activity in our study. The reasons of such discrepancies are unclear, but they could be linked to the high concentrations of pyrethroids used in some previous studies [26] or to the different methodological approaches retained for investigating ABC efflux pump, *i*.*e*., ATPase activity versus effective transport of reference substrates.

Finally, the hypothesis that pyrethroids may alter expression of drug transporters, notably in the liver, has likely to be additionally considered. Indeed, pyrethroids can activate drug sensing receptors like PXR and CAR [17, 18], which are well-known to be implicated in drug transporter expression regulation [79, 80]. Activation of PXR or CAR by pyrethroids may consequently result in up-regulation of transporters targeted by these receptors such as P-gp [81], MRP2 [82] or MRP3 [83]. Whether such putative regulations may occur in response to environmental concentrations of pyrethroids and may have functional consequences would deserve further studies.

In summary, the pyrethroids allethrin and tetramethrin were found to inhibit activity of various ABC and SLC drug transporters. Such inhibitions of drug transporters however occurred for concentrations of the two pyrethroids much higher than those commonly expected in response to environmental exposure, making unlikely any relevant contribution of transporter inhibition to pyrethroid toxicity in environmentally exposed humans.

## Supporting Information

S1 File List of molecular descriptors correlated with OCT1 activity inhibition by pyrethroids ((|r|>0.80). (XLSX)

S1 Table Number of possible stereoisomers for pyrethroids. (DOCX)

S2 Table Parameters for transporter-expressing cell culture. (DOCX)

S3 Table Parameters of drug transport assays. (DOCX)

S4 Table Molecular descriptors highly correlated with OCT1 activity inhibition by pyrethroids ((|r|>0.92). (DOCX)

S1 Fig Chemical structures of pyrethroids. (TIF)

S2 Fig ABC transporter activities in ABC transporter-expressing reference cell lines. P-gp, MRP2 and BCRP activities were determined through measuring cellular accumulation (P-gp and MRP2 activities) or retention (BCRP activity) of reference substrates (rhodamine 123 for P-gp, CF for MRP2 and Hoechst 33342 for BCRP), in the absence or presence of reference inhibitors (50 μM verapamil for P-gp, 2 mM probenecid for MRP2 and 10 μM fumitremorgin C for BCRP), in parental MCF7 and P-gp-positive MCF7R cells, in MRP2-positive HuH-7 cells and in HEK-BCRP cells. Data shown are the means ± SEM of at least three independent assays, each being performed in triplicate. *, p<0.05. FAU, fluorescence arbitrary unit.
(TIF)

S3 Fig Concentration-dependent effects of allethrin and tetramethrin towards MRP2 and BCRP activities. (A) MRP2 and (B) BCRP activities were determined in the absence or presence of various concentrations of allethrin or tetramethrin, as described in Materials and Methods. Data are expressed as percentages of transporter activity found in control untreated cells, arbitrarily set at 100%; they are the means ± SEM of three independent assays, each being performed in triplicate. IC_50_ values are indicated at the top of graphs.
(TIF)

S4 Fig OATP/OAT activities in transporter-expressing reference cell clones. OATP1B1, OATP1B3, OATP2B1, OAT1 and OAT3 activities were determined through measuring intracellular accumulation of reference substrates (E3S for OATP1B1 and OATP2B1 and fluorescein for OATP1B3, OAT1 and OAT3), in the absence or presence of the reference inhibitor probenecid, in OATP- or OAT-transfected cells and in control parental transporter-untransfected cells. Data shown are the means ± SEM of at least three independent assays, each being performed in triplicate. *, p<0.05. FAU, fluorescence arbitrary unit.
(TIF)

S5 Fig Concentration-dependent effects of allethrin and tetramethrin towards OATP1B1, OATP2B1 and OAT3 activities. (A) OATP1B1, (B) OATP2B1 and (C) OAT3 activities were determined in the absence or presence of various concentrations of allethrin or tetramethrin, as described in Materials and Methods. Data are expressed as percentages of transporter activity found in control untreated cells, arbitrarily set at 100%; they are the means ± SEM of three independent assays, each being performed in triplicate. (A, C) IC_50_ and (B) EC_50_ values are indicated at the top of graphs.
(TIF)

S6 Fig MATE/OCT activities in transporter-expressing reference cell clones. MATE1, MATE2-K, OCT1 and OCT2 activities were determined through measuring intracellular accumulation of reference substrates (TEA for MATE1 and MATE2-K, DAPI for OCT1 and rhodamine 123 for OCT2), in the absence or presence of reference inhibitors (verapamil for MATEs and OCT1 and amitriptyline for OCT2), in MATE- or OCT-transfected HEK293 cells and in control HEK-MOCK cells. Data shown are the means ± SEM of at least three independent assays, each being usually performed in triplicate. *, p<0.05. FAU, fluorescence arbitrary unit.
(TIF)

S7 Fig Concentration-dependent effects of allethrin and tetramethrin towards MATE1, OCT1 and OCT2 activities. (A) MATE1, (B) OCT1 and (C) OCT2 activities were determined in the absence or presence of various concentrations of allethrin or tetramethrin, as described in Materials and Methods. Data are expressed as percentages of transporter activity found in control untreated cells, arbitrarily set at 100%; they are the means ± SEM of three independent assays, each being performed in triplicate. IC_50_ values are indicated at the top of graphs.
(TIF)

S8 Fig Linear regression analysis of percentage of OCT1 activity inhibition by pyrethroids versus molecular descriptor values. Linear regression analysis was performed for percentages of OCT1 activity inhibition versus values of the molecular descriptors piPC02, piPC03, piPC04, piPC05, piPC06, SpDiam_X, JGI3, SM02_EA(bo) and SM04_EA(bo). The r^2^ value, a measure of the goodness of the fit, and the *p*-value are indicated at the top of graphs.
(TIF)
