# Supplementary material for: Inhibition of Human Drug Transporter Activities by the Pyrethroid Pesticides Allethrin and Tetramethrin
Source: PLoS One. 2017 Jan 18;12(1):e0169480. doi: 10.1371/journal.pone.0169480 (PMC5242521; doi:10.1371/journal.pone.0169480)
Supplement: S1 Table — (DOCX) [file pone.0169480.s002.docx]

| **Pyrethroid** | **Number of chiral carbons** | **Number of possible stereoisomers** |
| --- | --- | --- |
| Allethrin | 3 | 8 |
| Bifenthrin | 2 | 4 |
| β-Cyfluthrin | 1 | 2 |
| λ-Cyhalothrin | 1 | 2 |
| β-Cypermethrin | 2^a^ | 4^a^ |
| Deltamethrin | 3 | 8^b^ |
| Esfenvalerate | 2 | 4^c^ |
| Fenproprathin | 1 | 2 |
| τ-Fluvalinate | 1^d^ | 2^d^ |
| *cis*-Permethrin | 1^e^ | 2^e^ |
| *trans*-Permethrin | 1^e^ | 2^e^ |
| Resmethrin | 2 | 4 |
| Tefluthrin | 2 | 4^f^ |
| Tetramethrin | 2 | 4 |

^a^Cypermethrin contains 3 chiral carbons and corresponds to 8 possible stereoisomers

^b^Technical deltamethrin preparation contains only one stereoisomer [33]

^c^Esfenvalerate is comprised of 84% of one stereoisomer (SS configuration) [33]

^d^Fluvalinate contains 2 chiral carbons and corresponds to 4 possible stereoisomers

^e^Permethrin contains 2 chiral carbons and corresponds to 4 possible stereoisomers

^f^Technical tefluthrin preparation is a mixture of only two cis-stereoisomers [33]
