# Supplementary material for: Inhibition of Human Drug Transporter Activities by the Pyrethroid Pesticides Allethrin and Tetramethrin
Source: PLoS One. 2017 Jan 18;12(1):e0169480. doi: 10.1371/journal.pone.0169480 (PMC5242521; doi:10.1371/journal.pone.0169480)
Supplement: S2 Table — (DOCX) [file pone.0169480.s003.docx]

| **Cells** | **Nature of cell culture surface** | **Initial cell seeding/well^a^** | **Number of days in culture before performing transporter assays** |
| --- | --- | --- | --- |
| MCF7R | polystyrene | 80000 | 3 |
| HuH-7 | polystyrene | 70000 | 3 |
| HEK-MOCK | poly-D-lysine | 40000 | 5 |
| HEK-BCRP | poly-D-lysine | 110000 | 3 |
| HEK-OCT1 | poly-D-lysine | 50000 | 5 |
| HEK-OCT2 | poly-D-lysine | 40000 | 5 |
| HEK-OAT1 | poly-D-lysine | 50000 | 5 |
| HEK-OAT3 | poly-D-lysine | 30000 | 5 |
| HEK-MATE1 | poly-D-lysine | 30000 | 5 |
| HEK-MATE2-K | poly-D-lysine | 40000 | 5 |
| HEK-OATP2B1 | poly-D-lysine | 40000 | 5 |
| CHO wild-type | polystyrene | 15000 | 5 |
| CHO-OATP1B1 | polystyrene | 15000 | 5 |
| CHO-OATP1B3 | polystyrene | 18000 | 5 |

^a^Cells were plated in 48-multiwell plates
