# Supplementary material for: Inhibition of Human Drug Transporter Activities by the Pyrethroid Pesticides Allethrin and Tetramethrin
Source: PLoS One. 2017 Jan 18;12(1):e0169480. doi: 10.1371/journal.pone.0169480 (PMC5242521; doi:10.1371/journal.pone.0169480)
Supplement: S3 Table — (DOCX) [file pone.0169480.s004.docx]

| **Cells** | **Transporter** | **Substrate** | **Reference inhibitor** | **Incubation time** |
| --- | --- | --- | --- | --- |
| MCF7R | P-gp | Rhodamine 123  (5.3 μM) | Verapamil  (50 μM) | 30 min |
| HuH-7 | MRP2 | CF^a^  (3.0 μM) | Probenecid (2 mM) | 30 min |
| HEK-BCRP | BCRP | Hoechst 33342 (16.2 μM) | Fumitremorgin C  (10 μM) | 30 min  (+ 90 min efflux) |
| HEK-OCT1 | OCT1 | DAPI  (1.0 μM) | Verapamil (100 μM) | 5 min |
| HEK-OCT2 | OCT2 | Rhodamine 123  (1.0 µM) | Amitriptyline  (100 µM) | 5 min |
| HEK-OAT1 | OAT1 | Fluorescein  (10.0 µM) | Probenecid  (1 mM) | 5 min |
| HEK-OAT3 | OAT3 | Fluorescein  (10.0 µM) | Probenecid (1 mM) | 5 min |
| HEK-MATE1 | MATE1 | [^14^C]-TEA (29.0 µM) | Verapamil  (100 µM) | 5 min |
| HEK-MATE2-K | MATE2K | [^14^C]-TEA  (29.0 µM) | Verapamil  (200 µM) | 5 min |
| CHO-OATP1B1 | OATP1B1 | [^3^H]-E3S (3.7 nM) | Probenecid  (10 mM) | 5 min |
| CHO-OATP1B3 | OATP1B3 | Fluorescein  (10.0 μM) | Probenecid (10 mM) | 5 min |
| HEK-OATP2B1 | OATP2B1 | [^3^H]-E3S  (3.7 nM) | Probenecid  (10 mM) | 5 min |

^a^ CF is used under its non-fluorescent diacetate form for cell incubation.
