# Supplementary material for: Inhibition of Human Drug Transporter Activities by the Pyrethroid Pesticides Allethrin and Tetramethrin
Source: PLoS One. 2017 Jan 18;12(1):e0169480. doi: 10.1371/journal.pone.0169480 (PMC5242521; doi:10.1371/journal.pone.0169480)
Supplement: S4 Table — (DOCX) [file pone.0169480.s005.docx]

| **Block description** | **Descriptor ID** | **Significance** | **r** | ***p*-value** |
| --- | --- | --- | --- | --- |
| Walk and path counts | piPC02 | molecular multiple path count of order 2 | - 0.9325 | < 0.0001 |
|  | piPC03 | molecular multiple path count of order 3 | -0.9275 | < 0.0001 |
|  | piPC04 | molecular multiple path count of order 4 | -0.9258 | < 0.0001 |
|  | piPC05 | molecular multiple path count of order 5 | -0.946 | < 0.0001 |
|  | piCO6 | molecular multiple path count of order 6 | -0.9406 | < 0.0001 |
| 2D matrix-based descriptors | SpDiam_X | spectral diameter from chi matrix | -0.9321 | <0.0001 |
| 2D autocorrelations | JGI3 | mean topological charge index of order 3 | 0.9368 | <0.0001 |
| Edge adjacency indices | SM02_EA(bo) | spectral moment of order 2 from edge adjacency mat. weighted by bond order | -0,9321 | <0.0001 |
|  | SM04_EA(bo) | spectral moment of order 4 from edge adjacency mat. weighted by bond order | -0,9255 | <0.0001 |
