# Supplementary figures and images for: Inhibition of Human Drug Transporter Activities by the Pyrethroid Pesticides Allethrin and Tetramethrin
Source: PLoS One. 2017 Jan 18;12(1):e0169480. doi: 10.1371/journal.pone.0169480 (PMC5242521; doi:10.1371/journal.pone.0169480)

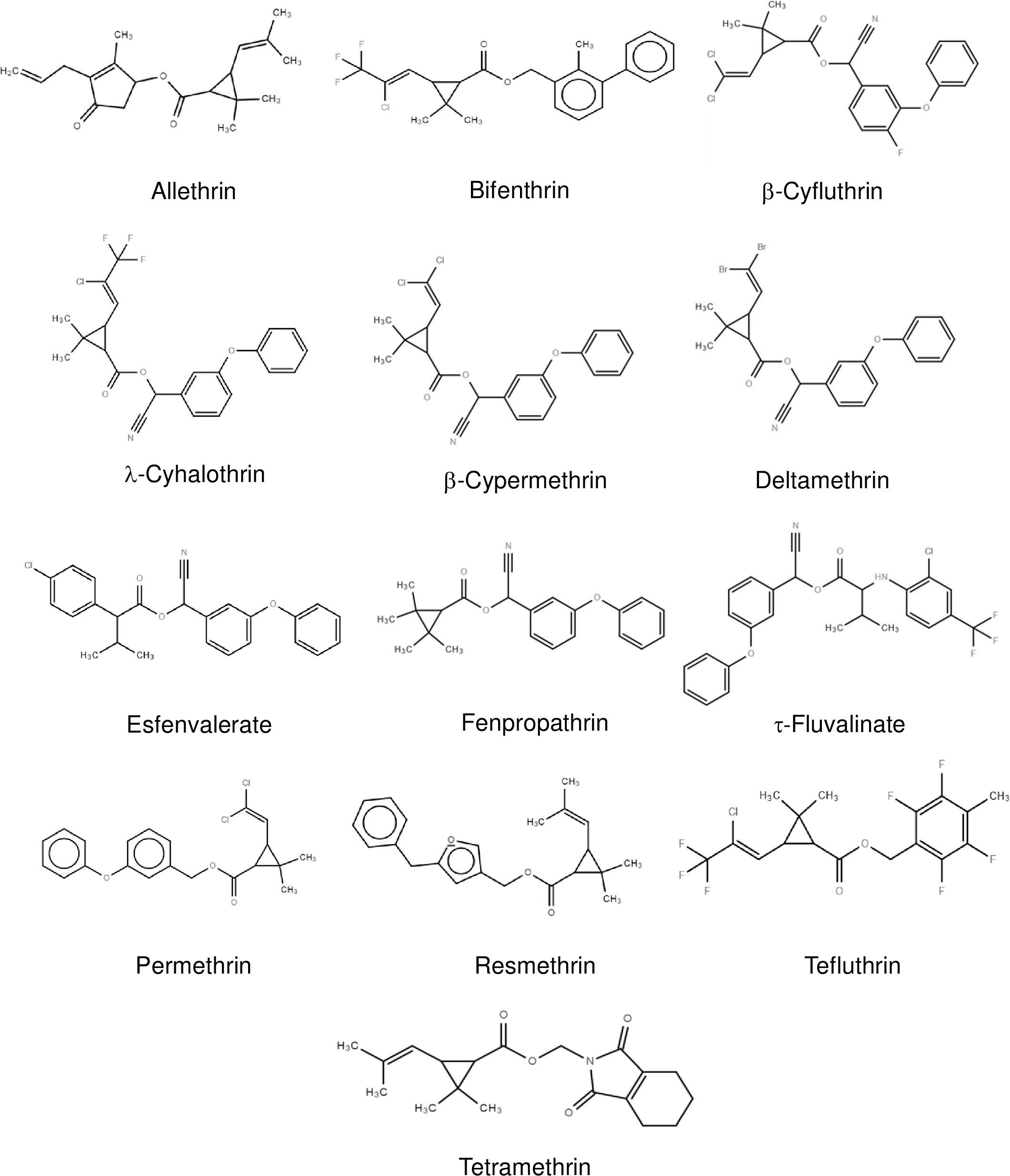

Supplement: S1 Fig — (TIF) [file pone.0169480.s006.tif]

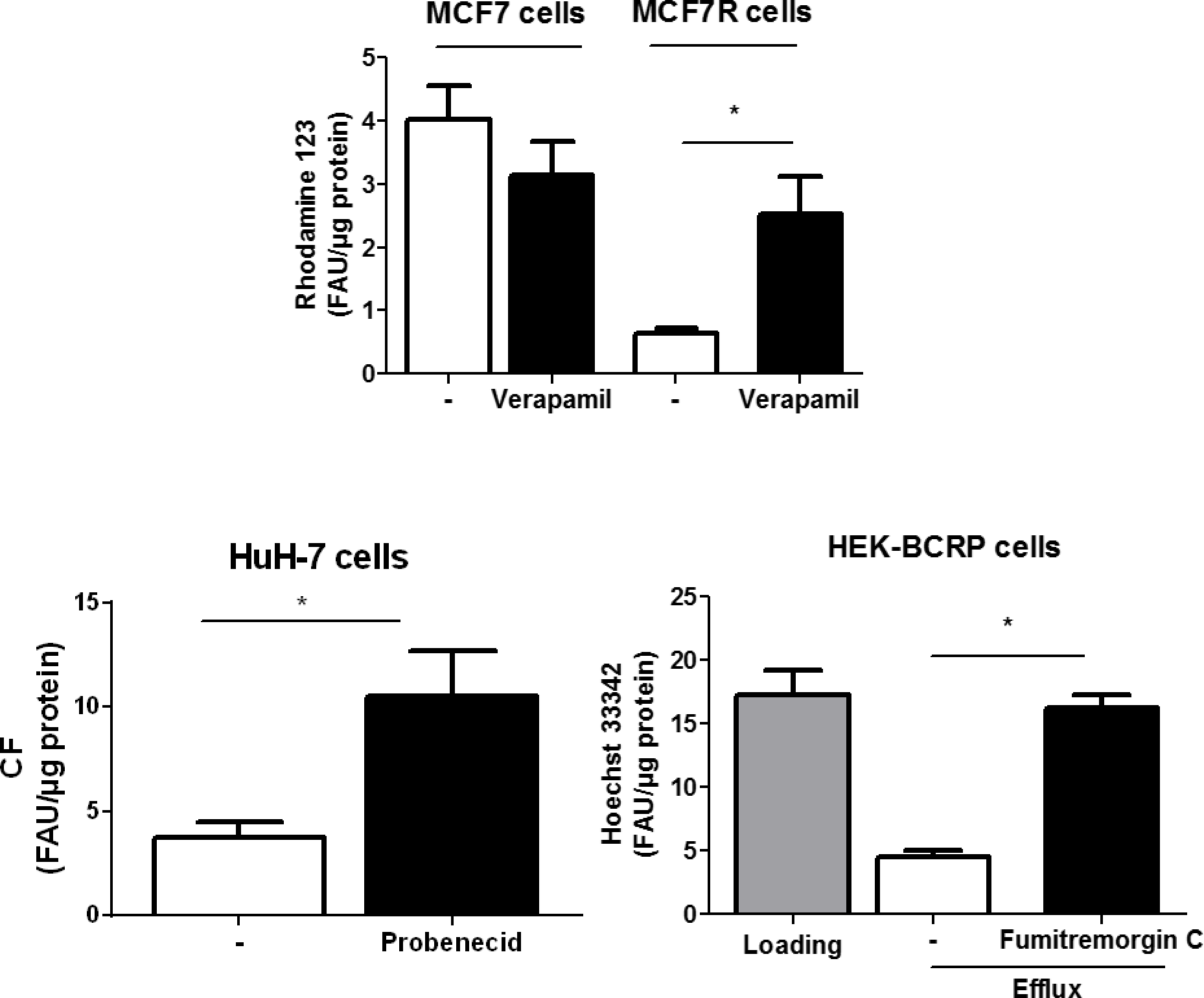

Supplement: S2 Fig — P-gp, MRP2 and BCRP activities were determined through measuring cellular accumulation (P-gp and MRP2 activities) or retention (BCRP activity) of reference substrates (rhodamine 123 for P-gp, CF for MRP2 and Hoechst 33342 for BCRP), in the absence or presence of reference inhibitors (50 μM verapamil for P-gp, 2 mM probenecid for MRP2 and 10 μM fumitremorgin C for BCRP), in parental MCF7 and P-gp-positive MCF7R cells, in MRP2-positive HuH-7 cells and in HEK-BCRP cells. Data shown are the means ± SEM of at least three independent assays, each being performed in triplicate. *, p<0.05. FAU, fluorescence arbitrary unit. (TIF) [file pone.0169480.s007.tif]

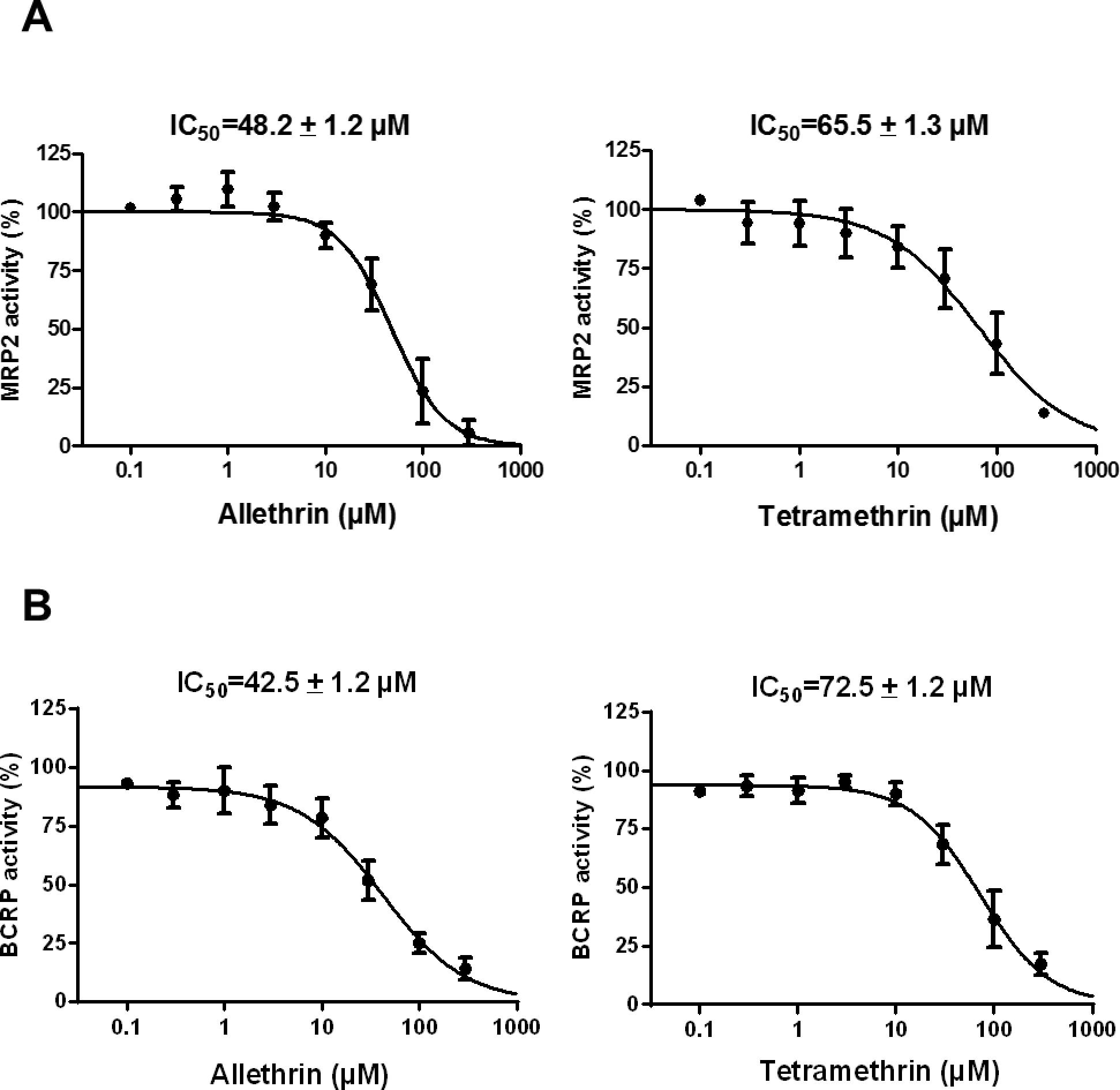

Supplement: S3 Fig — (A) MRP2 and (B) BCRP activities were determined in the absence or presence of various concentrations of allethrin or tetramethrin, as described in Materials and Methods. Data are expressed as percentages of transporter activity found in control untreated cells, arbitrarily set at 100%; they are the means ± SEM of three independent assays, each being performed in triplicate. IC50 values are indicated at the top of graphs. (TIF) [file pone.0169480.s008.tif]

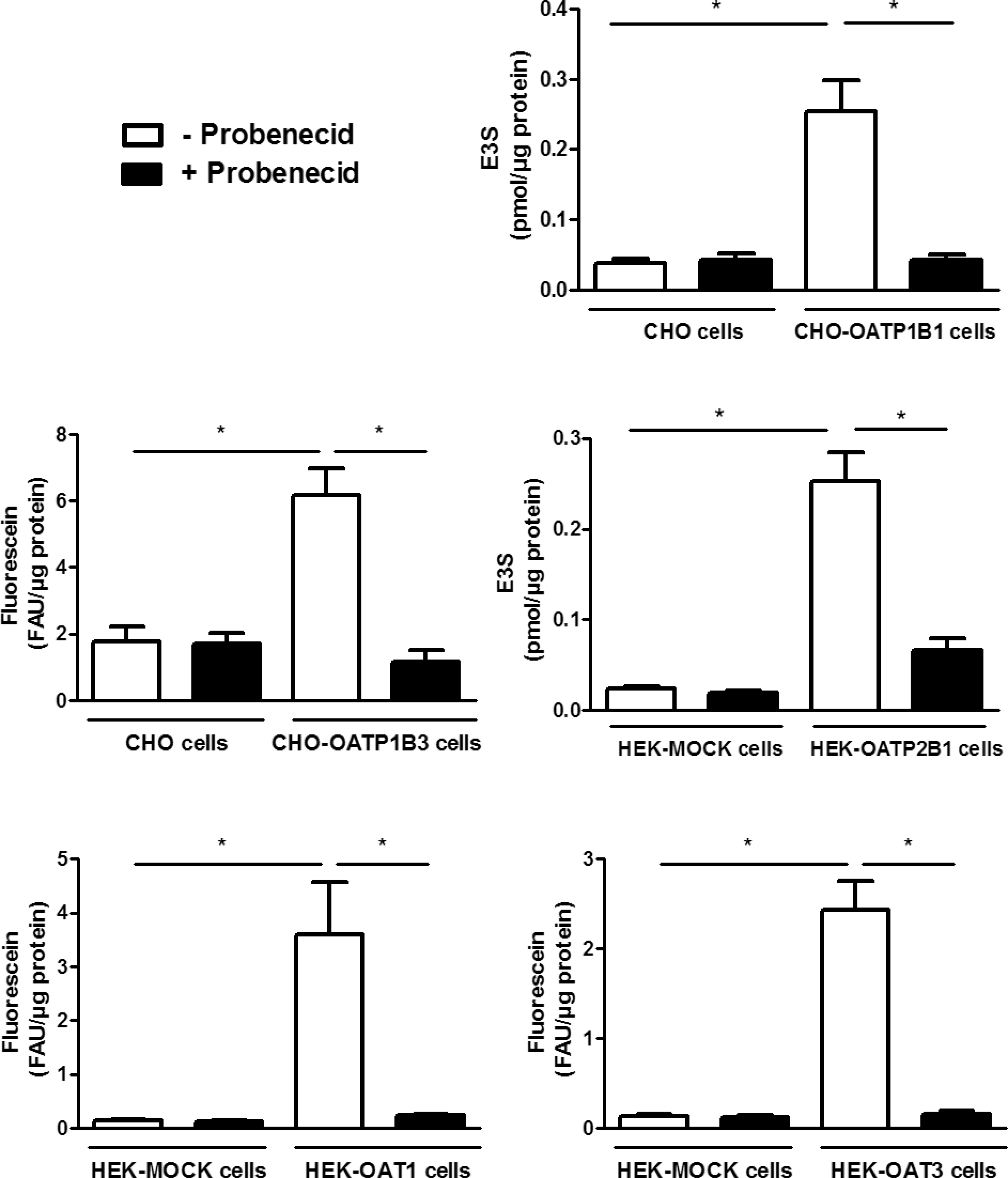

Supplement: S4 Fig — OATP1B1, OATP1B3, OATP2B1, OAT1 and OAT3 activities were determined through measuring intracellular accumulation of reference substrates (E3S for OATP1B1 and OATP2B1 and fluorescein for OATP1B3, OAT1 and OAT3), in the absence or presence of the reference inhibitor probenecid, in OATP- or OAT-transfected cells and in control parental transporter-untransfected cells. Data shown are the means ± SEM of at least three independent assays, each being performed in triplicate. *, p<0.05. FAU, fluorescence arbitrary unit. (TIF) [file pone.0169480.s009.tif]

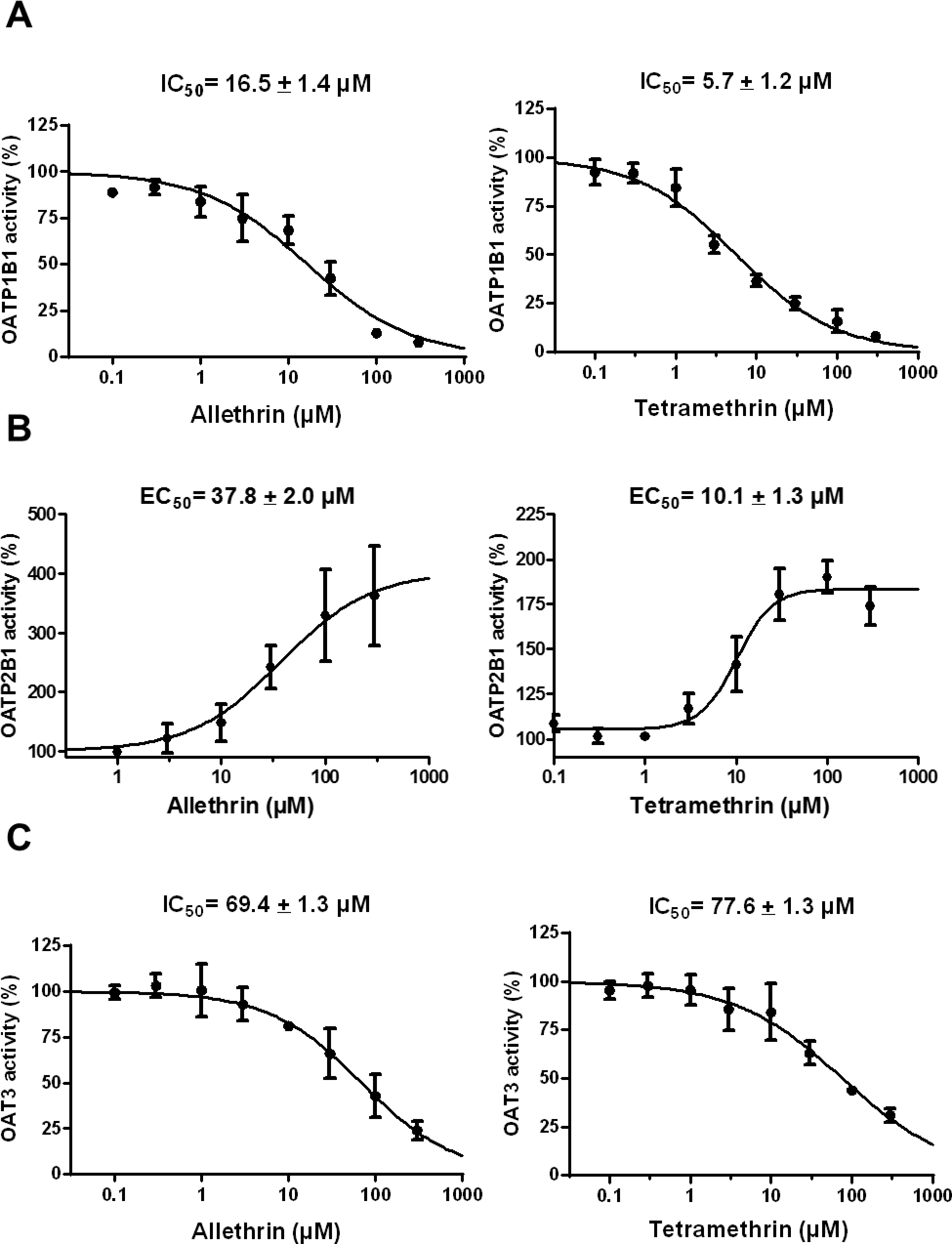

Supplement: S5 Fig — (A) OATP1B1, (B) OATP2B1 and (C) OAT3 activities were determined in the absence or presence of various concentrations of allethrin or tetramethrin, as described in Materials and Methods. Data are expressed as percentages of transporter activity found in control untreated cells, arbitrarily set at 100%; they are the means ± SEM of three independent assays, each being performed in triplicate. (A, C) IC50 and (B) EC50 values are indicated at the top of graphs. (TIF) [file pone.0169480.s010.tif]

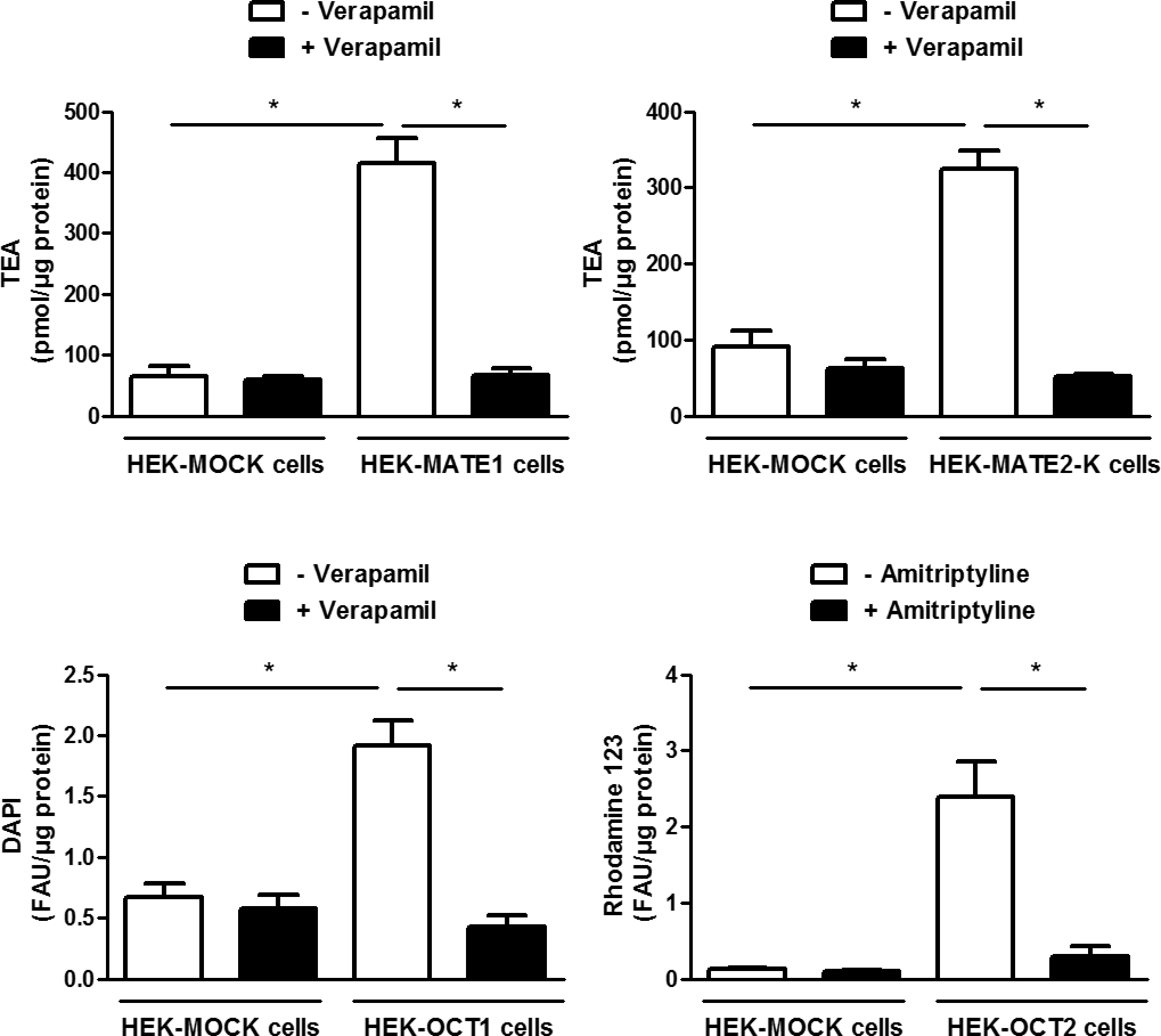

Supplement: S6 Fig — MATE1, MATE2-K, OCT1 and OCT2 activities were determined through measuring intracellular accumulation of reference substrates (TEA for MATE1 and MATE2-K, DAPI for OCT1 and rhodamine 123 for OCT2), in the absence or presence of reference inhibitors (verapamil for MATEs and OCT1 and amitriptyline for OCT2), in MATE- or OCT-transfected HEK293 cells and in control HEK-MOCK cells. Data shown are the means ± SEM of at least three independent assays, each being usually performed in triplicate. *, p<0.05. FAU, fluorescence arbitrary unit. (TIF) [file pone.0169480.s011.tif]

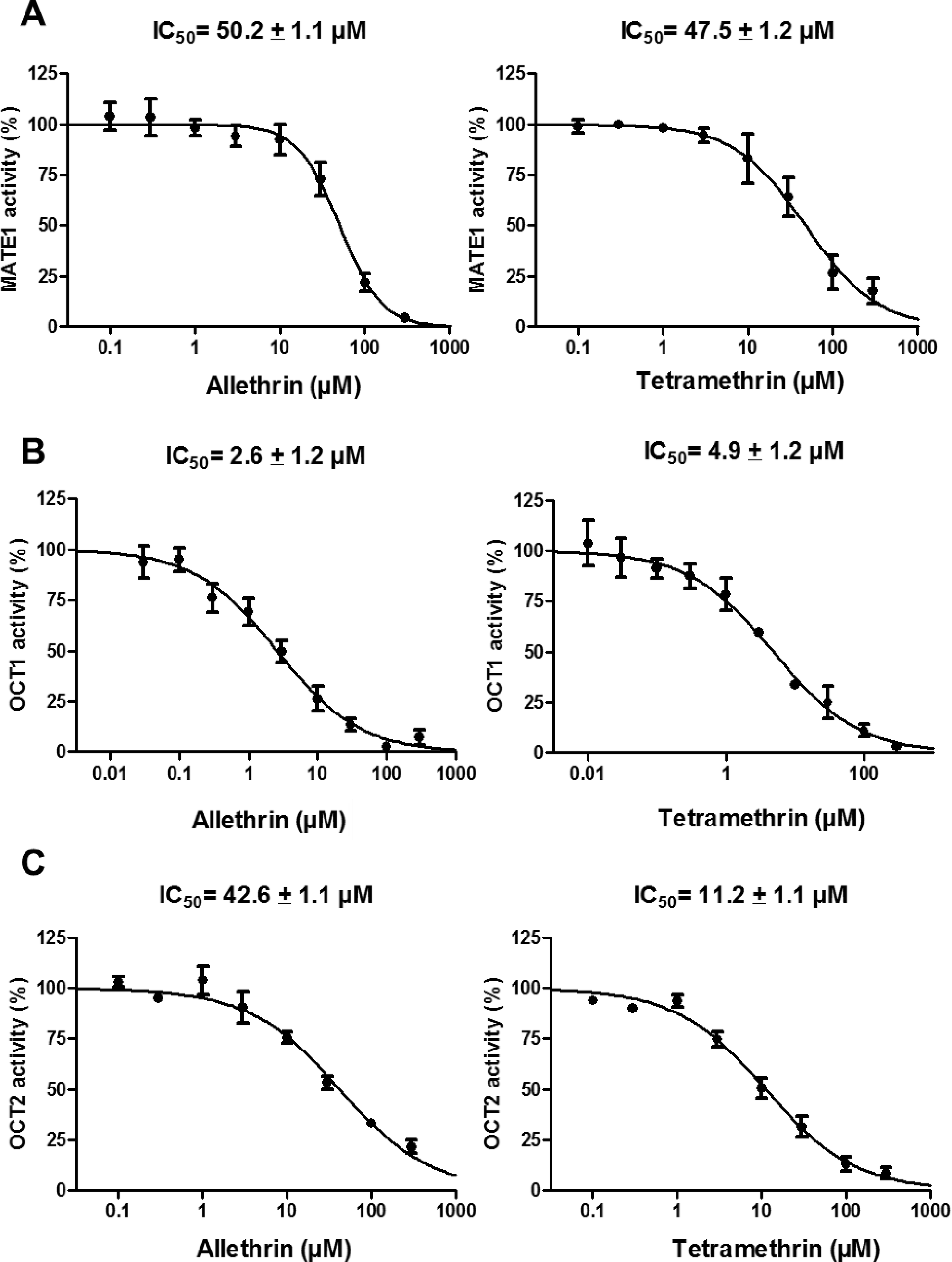

Supplement: S7 Fig — (A) MATE1, (B) OCT1 and (C) OCT2 activities were determined in the absence or presence of various concentrations of allethrin or tetramethrin, as described in Materials and Methods. Data are expressed as percentages of transporter activity found in control untreated cells, arbitrarily set at 100%; they are the means ± SEM of three independent assays, each being performed in triplicate. IC50 values are indicated at the top of graphs. (TIF) [file pone.0169480.s012.tif]

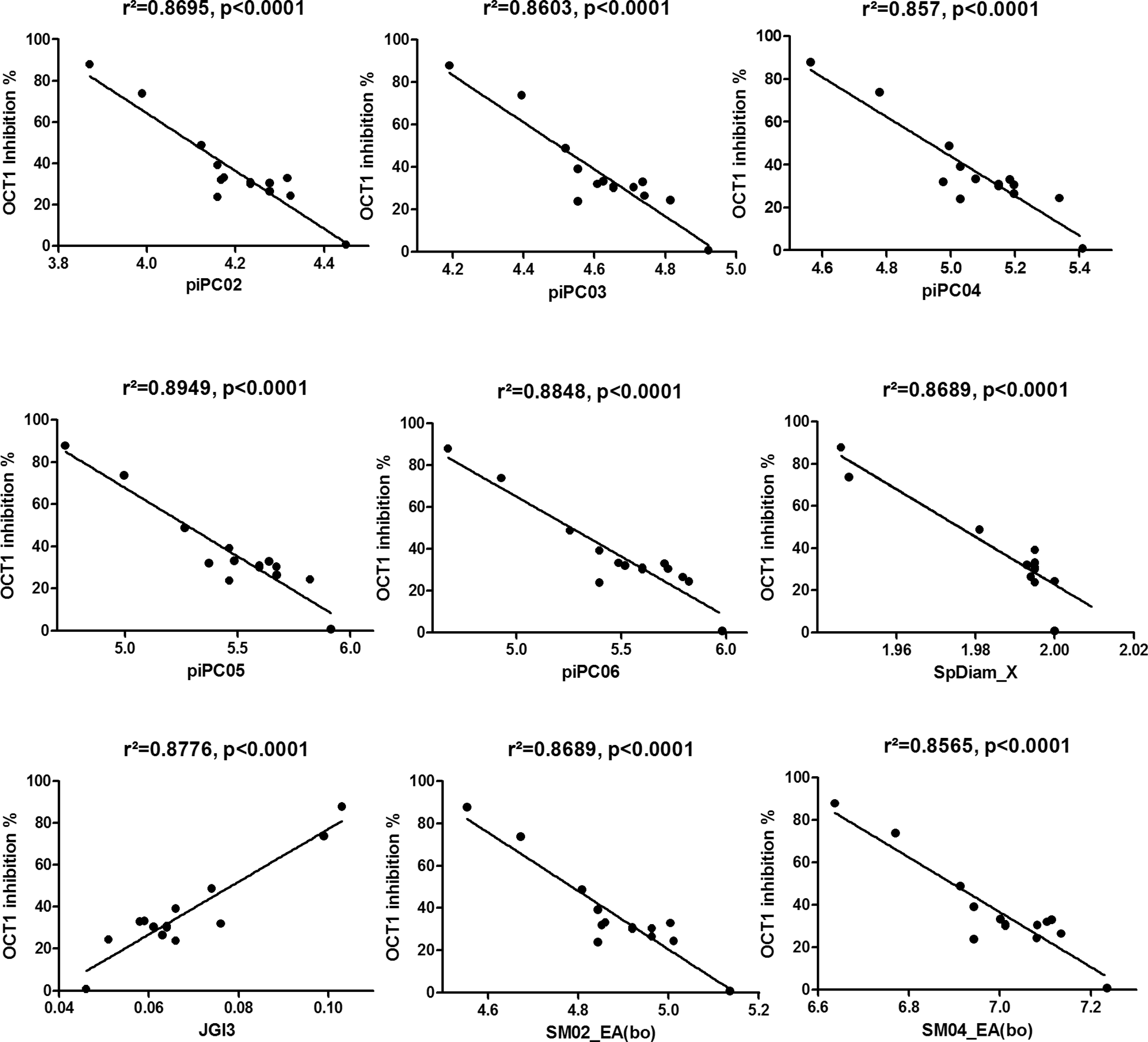

Supplement: S8 Fig — Linear regression analysis was performed for percentages of OCT1 activity inhibition versus values of the molecular descriptors piPC02, piPC03, piPC04, piPC05, piPC06, SpDiam_X, JGI3, SM02_EA(bo) and SM04_EA(bo). The r2 value, a measure of the goodness of the fit, and the p-value are indicated at the top of graphs. (TIF) [file pone.0169480.s013.tif]
